# Shaping the Gradient: Ether-Type Polar Modifiers for the Statistical Anionic Copolymerization of Styrene and Isoprene

**DOI:** 10.1021/acsmacrolett.6c00201

**Published:** 2026-05-28

**Authors:** Thi Dinh, Marvin Steube, Tobias Johann, Holger Frey, Axel H. E. Müller

**Affiliations:** Department of Chemistry, 9182 Johannes Gutenberg-University Mainz, Duesbergweg 10-14, D-55128 Mainz, Germany

## Abstract

Living anionic copolymerization of isoprene and styrene, initiated by alkyllithium compounds in nonpolar solvent, typically yields commercially relevant tapered copolymers, widely used as thermoplastic elastomers. Lewis base modifiers enable both gradient and microstructure control. Ether-type ligands with varying coordination number were systematically investigated in styrene/isoprene copolymerization in cyclohexane using *in situ* near-infrared spectroscopy, focusing on their electronic and steric effects and coordination behavior. Pronounced changes in reactivity ratios (*r*_I_ ≫ *r*_S_ to *r*_I_ ≪ *r*_S_) enabled access to tapered, gradient, inverse-gradient, and inverse-tapered architectures with increasing modifier strength. NMR analysis revealed systematic polyisoprene (PI) microstructural variations, leading to increased vinyl (1,2- and 3,4-PI) content. In summary, the most promising bidentate modifier, 2,2-di­(2-tetrahydrofuryl)­propane (DTHFP), provides efficient architectural tuning by changing the modifier/lithium ratio, yielding random copolymers at concentrations as low as 0.25 equiv with respect to the *sec*-BuLi-initiator employed.

## Graphical abstract

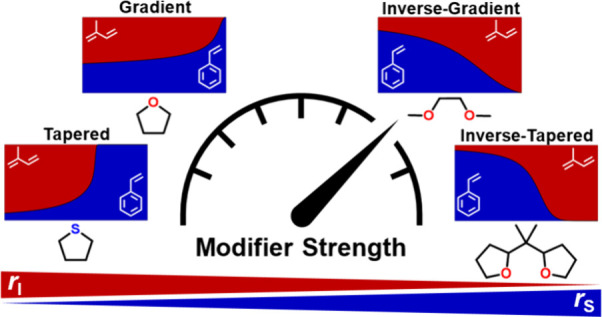

Living anionic polymerization enables the synthesis of well-defined styrene/diene copolymers with precise control over molecular weight, dispersity, composition, and architecture, and is established in industrial thermoplastic elastomer (TPE) production.^1–7^ TPEs are typically ABA-type block copolymers, consisting of glassy polystyrene endblocks (high glass transition temperature, *T*_g_) and a soft (low *T*_g_) poly­(1,3-diene) midblock commonly derived from butadiene (B) or isoprene (I).^4,8–12^ In nonpolar solvents, living chain ends exist in equilibrium between inactive aggregated and active nonaggregated species, strongly affecting copolymerization kinetics and comonomer incorporation.^13–18^ Initiation with *sec*-butyllithium (*s*-BuLi) in cyclohexane (CyH) yields highly disparate reactivity ratios of styrene (S) and 1,3-dienes (*r*_B_ = 15; *r*_S_ = 0.05 and *r*_I_ = 10.1; *r*_S_ = 0.013) primarily governed by differences in the crossover propagation rates.^13,14,17,19,20^ Preferential early incorporation of diene units results in so-called ‘tapered’ copolymers displaying a steep gradient.^6,7,10^ Disruption of chain-end aggregates by polar additives, so-called polar modifiers leading to di- and monoetherate complexes, increases chain-end reactivity, inverts reactivity ratios, and enables ‘inverse-tapered’ or random architectures through systematic variation of the modifier-to-initiator ratio.^14,21–26^

Polar modifiers, investigated in the homo- and copolymerization of styrene and 1,3-dienes are either Lewis acid (π-type) ligands, such as alkali alkoxides,^27–29^ or Lewis base (σ-type) ligands, like amines and ethers, in particular triethylamine,^26^ tetramethylethylenediamine,^30^ tetrahydrofuran (THF),^21–23,31^ and 2,2-di­(2-tetrahydrofuryl)­propane (DTHFP).^22,32–34^ While all modifiers profoundly affect chain-end aggregation and polymerization kinetics in distinct ways, they significantly differ in their effect on comonomer sequence, polyisoprene microstructure, and resulting material properties. Ether-type modifiers promote 1,2- and 3,4-addition at the expense of 1,4-addition with increasing modifier content and coordinating strength (e.g., chelate effect of DTHFP compared to THF). This change in microstructure enables functionalization of the increased vinyl side chains, however it also increases the glass transition temperature of the TPE soft segment, and potentially compromises elastomeric performance.^21,22,25,35–37^ Consequently, balancing architectural control with microstructural integrity remains a central challenge in the rational design of styrenic TPEs via anionic polymerization. To date, most ether modifiers have been studied either in homopolymerizations or in copolymerizations under varying conditions, including different comonomer systems and modifier loadings.^21,22,24,26,30,36,38–41^ Such variations in experimental design, investigation method and structural characterization impede a direct comparison and systematic evaluation of modifier strength and the associated structure–property relationships.

In this work, the effect of a large variety of monodentate, bi- and polydentate ethers on the statistical anionic copolymerization of isoprene and styrene has been systematically investigated. Fast *in situ* near-infrared (NIR) spectroscopy, well-suited for rapid copolymerizations induced by polar additives, was employed to study copolymerization kinetics and reactivity ratios. Furthermore, the resulting effects on comonomer sequence, gradient profile, polyisoprene microstructure, and thermal properties are comprehensively analyzed.

A range of commercially available ether-based polar modifiers (PM) was used to systematically investigate steric, electronic, and chelating effects on the formed comonomer gradient. As illustrated in Figure 1, the modifiers can be categorized by their coordination number (CN). Monodentate ethers (CN = 1) include THF, 2,5-dimethyltetrahydrofuran (DMTHF), 2-methyltetrahydrofuran (MTHF), 1,4-dioxane (DOX), and tetrahydrothiophene (THT). Bidentate ethers (CN = 2) comprise 2,2-di­(2-tetrahydrofuryl)­propane (DTHFP) and 1,2-dimethoxyethane (DME). Diethylene glycol dimethyl ether (diglyme), triethylene glycol dimethyl ether (triglyme), 12-crown-4 (12C4), and 15-crown-5 (15C5) are classified as polydentate (CN = 3–5). All copolymerizations were initiated with *sec*-butyllithium (*s*-BuLi) in cyclohexane at ∼20 °C. The corresponding recorded temperature profiles are displayed in Figures S22–S26. The reactions were monitored *in situ* by near-infrared (NIR) spectroscopy in the wavenumber range of *ν̃* = 5900–6250 cm^–1^. An equimolar monomer feed and constant initial monomer and initiator concentrations were maintained throughout all experiments. The polar modifier-to-initiator ratio was set to [PM]/[Li] = 2 equivalents (equiv), with DTHFP additionally studied in the range 0.25 to 2 equiv and THT up to 20 equiv. A detailed description is given in the Supporting Information, Chapters 1–3, and the experimental parameters and resulting polymer characteristics are summarized in Table 1 and Table S1.

**Figure 1:**
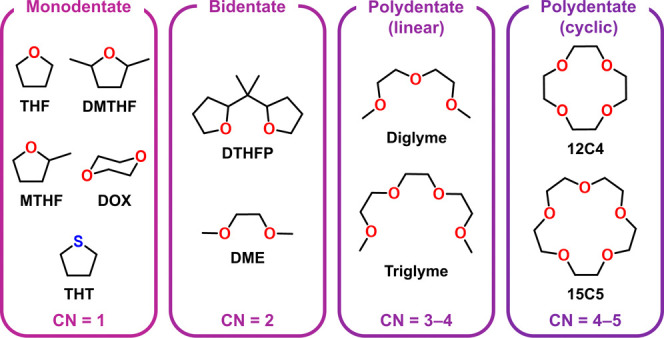
Polar modifiers classified according to their coordination number (CN).

**Table 1:**
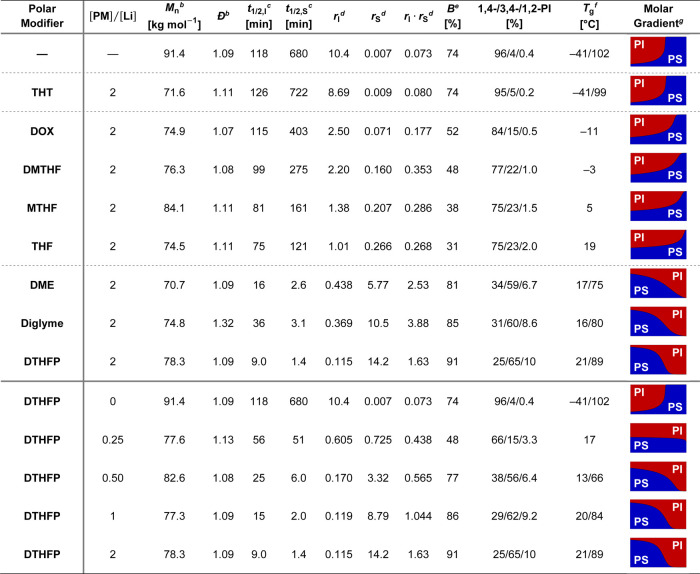
Experimental Conditions and Results of the Copolymerization of Styrene and Isoprene in Cyclohexane and in the Presence of Various Polar Modifiers, PM^a^

a Equimolar monomer feed of 50%_mol_ = 60%_wt_ = 57%_vol_ styrene, [S]_0_ = [I]_0_ = 0.65 mol L^–1^), [*s*-BuLi]_0_ = 1.44 mmol L^–1^, *T* = ∼20 °C.

b Molar masses, *M*_n_, and dispersities, *Đ*, determined via SEC (THF, PS-standards, RI-detector).

c Half-lives, *t*_1/2,I_ and *t*_1/2,S_, determined from time–conversion plots.

d Reactivity ratios, *r*_I_, *r*_S_ and *r*_I_ *r*_S_, determined from Meyer-Lowry fits.

e Blockiness, *B*, defined as the molar fraction of at least two consecutive (block-like) styrene units, and content of polyisoprene microstructure, 1,4-/3,4-/1,2-PI, determined from ^1^H- and *inverse-gated* ^13^C NMR spectroscopy (see SI, Chapter 5 and Figures S7–S21).

f Glass transition temperatures determined from DSC using the second heating curve (Figures S41–S45).

g Molar-based composition diagram as a plot of instantaneous styrene incorporation, *F*_S_, vs total monomer conversion to visualize copolymer gradient.

The corresponding SEC traces are presented in Figures S2–S6. NMR analysis (Figures S7–S21) confirmed the chemical composition of the obtained copolymers. Most synthesized copolymers reached the targeted molar mass of 80 kg mol^–1^ with low dispersities. However, the use of diglyme showed signs of termination reactions (Figure S1), leading to broader molar mass distributions and slightly incomplete monomer consumption. Even more pronounced effects were observed with CN = 3–5 modifiers, where high styrene incorporation is favored but isoprene polymerization became increasingly incomplete, resulting in lower molar masses. In the presence of crown ether 12C4, which is selective for lithium, conversion of both I and S was incomplete (Figure S1 and Table S1). However, while the conversion of isoprene is almost complete using diglyme (<98%) and nearly half complete using triglyme (∼46%), copolymerizations in the presence of crown ethers only led to low isoprene conversion (∼7–10%). The observed side reactions, occurring in the presence of modifiers based on ethylene glycol units (DME, diglyme, triglyme, 12C4, and 15C5), can be explained by the deprotonation of these units caused by the attack of the highly reactive, chelated polyisoprenyl anions, which is supposed as the main side reaction.^40,42^

The copolymerization monitoring using *in situ* NIR spectroscopy after deconvolution yielded individual time–conversion plots (Figures S29–S31) and plots of individual vs total conversion (Figures S32–S34). Representative plots for selected modifiers are also shown in Figure 2a,d and b,e. Due to fast styrene but incomplete isoprene conversion in the presence of triglyme, 12C4, and 15C5 (Figures S27–S28), the NIR spectra could not be evaluated to obtain kinetic plots.

**Figure 2:**
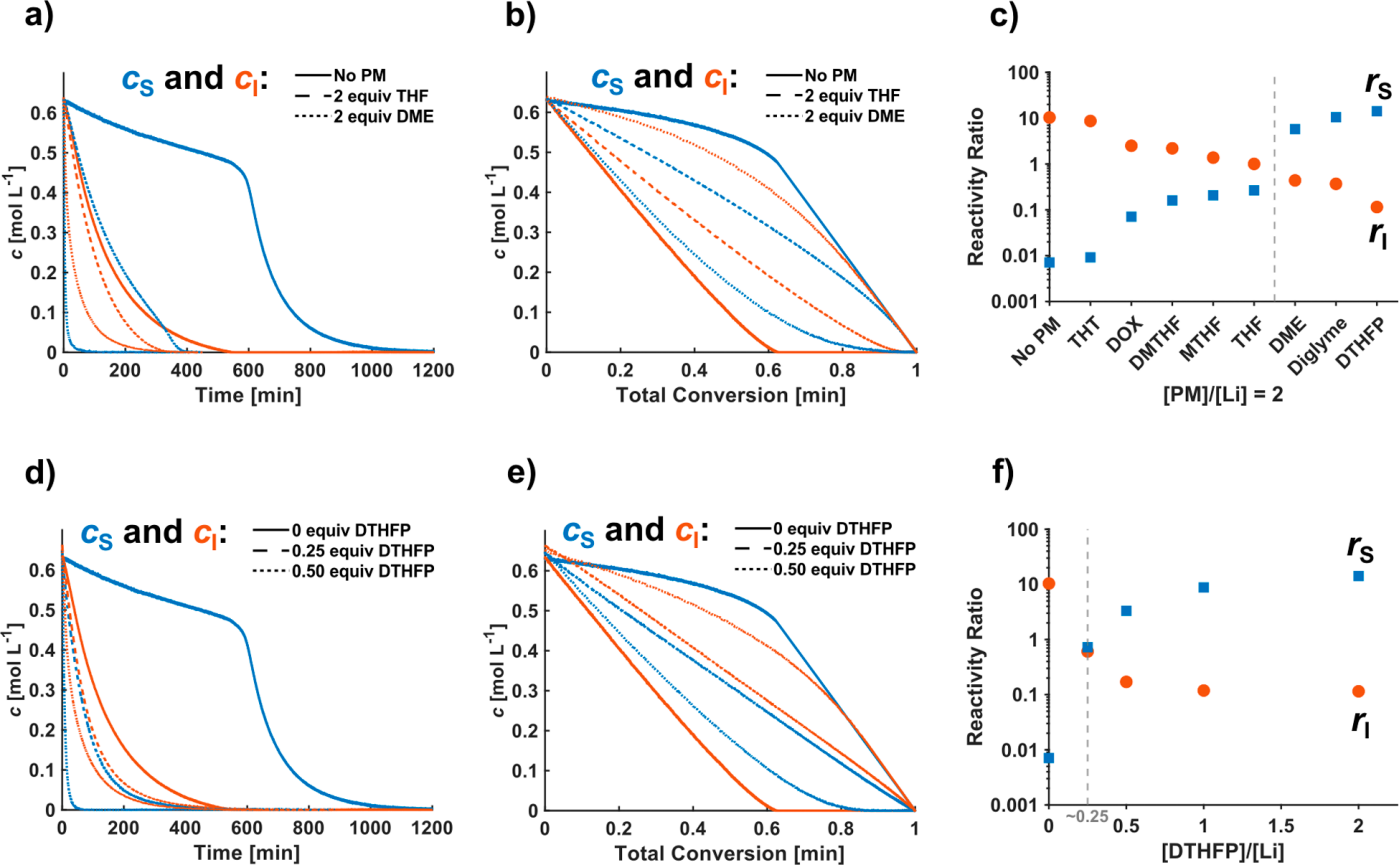
(a, d) Individual time–conversion plots, (b, e) individual vs total conversion plots of selected S/I copolymerizations and (c, f) reactivity ratios for [PM]/[Li] = 2 (top row) and various [DTHFP]/[Li] ratios (bottom row); with dashed lines separating monodentate PM from bi- and tridentate PMs (top row) or indicating a random architecture for 0.25 equiv DTHFP (bottom row).

In analogy to our previous works,^4,19,21^ the reactivity ratios (*r*_I_ = *k*_II_/*k*_IS_ and *r*_S_ = *k*_SS_/*k*_SI_) were calculated using the terminal model (nonlinear Meyer-Lowry fit; Figures S35–S37). This procedure is also recommended by IUPAC.^43^ The obtained values are given in Tables 1 and S1. In Figure 2c,f and Figure S48c, these reactivity ratios are plotted either as a function of the modifier type (top row) or as a function of increasing DTHFP content (bottom row). The molar-based copolymer gradient is visualized as the instantaneous styrene incorporation, *F*_S_, plotted as a function of total conversion (Table 1, Table S1, and Figure S46). The corresponding volume-based diagrams are presented in Figure S47. Since isoprene adds to PS-Li and PI-Li chain ends in three different regiochemistries, all reactivity ratios are averages of the regiochemical distributions of the isoprene segments.

For the series with a constant [PM]/[Li] ratio of 2, the modifiers were arranged with decreasing *r*_I_ (increasing *r*_S_), which corresponds to the estimated individual half-lives, *t*_1/2_ (see also Figure S49). The least effective monodentate modifiers THT and DOX, yield tapered architectures with PI-rich segments formed at early stages and a distinct PS block at high conversion (*r*_I_ ≫ *r*_S_). The modifiers DMTHF, MTHF, and THF, with *r*_I_ > *r*_S_, produce increasingly shallow gradients with less distinct PS block. A dramatic change is observed with bi- and polydentate modifiers, where reactivity ratios are inversed (*r*_I_ < *r*_S_), generating inverse-gradient architectures with DME and diglyme and an inverse-tapered architecture (*r*_I_ ≪ *r*_S_) with a pure PI end block using DTHFP. Notably, in the DTHFP series, even small modifier amounts are sufficient to produce nearly random (0.25 equiv) and inverse-tapered (0.5–2 equiv) copolymers, consistent with the work of Fuchs et al. on styrene/β-myrcene copolymerizations.^22^ In contrast, about 6 equiv of THF are needed to reach random copolymerization and 2500 equiv for the inverse-tapered architecture.^21^

The experimentally observed differences in reactivity ratios can be attributed solely to the nature of the Lewis bases employed, in particular their electronic and steric properties and coordination behavior. It turns out that for monodentate modifiers, this order perfectly corresponds to the Gutmann donor number, DN, of the Lewis bases,^44–46^ and a linear fit is obtained (Figure S50). The donor number concept fails for di- and polydentate ligands, as it does not take chelation with Li into account. Furthermore, DN cannot be used to compare different heteroatom-based modifiers. The thioether THT causes a comparatively small change in the reactivity ratio compared to the oxygen-containing ethers. This is attributed to the low polarity of the C—S bond (EN_S_ = 2.58; EN_C_ = 2.55; ΔEN = 0.03)^47^ compared to the C—O bond (EN_O_ = 3.45; EN_C_ = 2.55; ΔEN = 0.90).^47^ Consequently, the sulfur atom in THT is less electron-rich and functions as a weaker Lewis base. Even at 20 equiv the reactivity ratios remained essentially unchanged.

Among the monodentate oxygen-based modifiers, 1,4-dioxane (DN = 14.8 kcal mol^–1^)^48^ exhibits the weakest influence on the reactivity ratios, despite its two oxygen atoms. This may be unexpected but is explained by its behavior as a monodentate Lewis base, as the oxygen atoms in chair conformation are spatially too far apart to simultaneously coordinate to the same active chain end. In contrast, THF (DN = 20 kcal mol^–1^)^48^ reveals the strongest influence on the reactivity ratios and is thus the most effective monodentate modifier. The weaker effects observed for MTHF (DN = 18 kcal mol^–1^)^49^ and DMTHF are attributed to the presence of methyl substituents that introduce steric hindrance and limit efficient coordination to the lithium ion. The bidentate modifier DME interacts significantly more strongly with the lithium counterion than monodentate additives,^50^ due to chelation via both oxygens. The even stronger effect of DTHFP is explained by the perfect, preorganized chelation by both oxygens with minimal entropic cost, in contrast to the mobile methyl groups of DME. Contrary to expectation, the tridentate diglyme exhibits a weaker effect on reactivity ratios than the bidentate DTHFP. Although diglyme is highly flexible and represents a stronger Lewis base, it has no preorganization and must adopt a suitable conformation with loss of entropy.

Changes in reactivity ratios (corresponding to gradient profiles) are accompanied by pronounced variations in thermal behavior (Figure 3a,d and Figure S51a,d,g), as microphase separation is crucial for maintaining block-specific thermal properties. Copolymers with tapered or inverse-tapered gradients, resembling diblock architectures, display two *T*_g_ values associated with styrene- and isoprene-rich domains, respectively. For triglyme, 12C4, and 15C5, only the polystyrene *T*_g_ could be detected due to insufficient isoprene incorporation. In contrast, copolymers with flatter gradients or random sequence display only a single *T*_g_, reflecting the presence of a single mixed phase only. The observed *T*_g_ values and trends arise not only from the differences in comonomer sequence distribution and change in the gradient profile but also from the ‘blockiness’ and the regioisomeric composition of polyisoprene segments, which are given in Table 1 and Table S1 as well as in Figure 3 and Figure S51.

**Figure 3:**
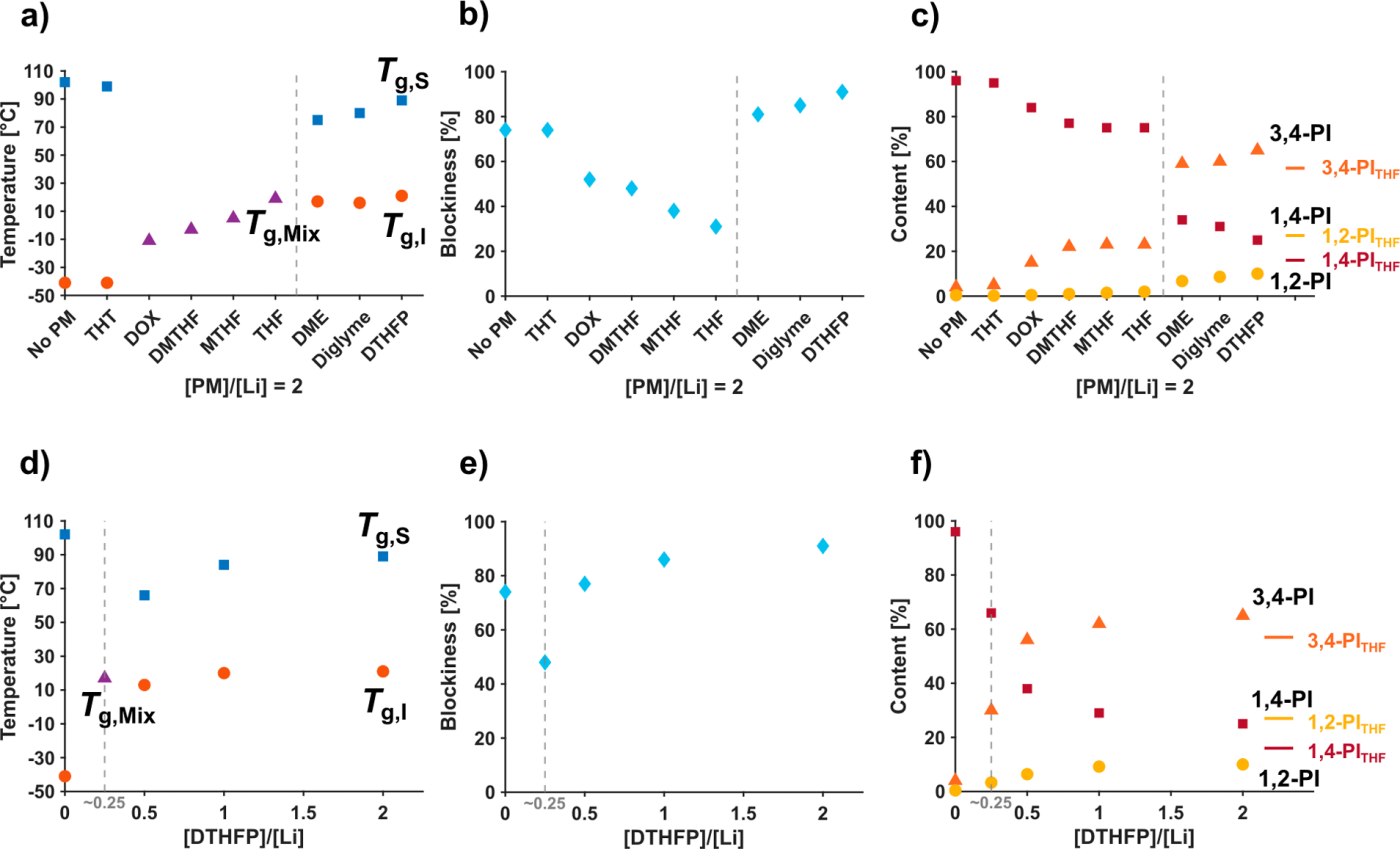
(a, d) Glass transition temperature (b, e) blockiness and (c, f) PI microstructure of P­(I-*co*-S) copolymers for [PM]/[Li] = 2 (top row) and various [DTHFP]/[Li] ratios (bottom row); with dashed lines separating monodentate PM from bi- and tridentate PMs (top row) or indicating a random architecture for 0.25 equiv DTHFP (bottom row), and violet triangles referring to *T*_g_ values of single mixed phases, *T*_g,Mix_.

The term ‘blockiness’ is defined as the fraction of two or more neighboring styrene segments as determined by ^1^H NMR (see Supporting Information, Chapter 5.1).^51^ Tapered and inverse-tapered copolymers exhibit high blockiness (73–96%), whereas random and flatter gradient copolymers display lower values (31–52%). The glass transition temperature of the styrene-rich phases, *T*_g,S_, remains high (99–102 °C) for copolymers synthesized in pure CyH and in the presence of THT, which is in good agreement with the literature.^21^ Lower *T*_g,S_ values (75–89 °C) are observed for the inverse-tapered copolymers obtained with di- and multidentate modifiers. Here, short isoprene segments interrupt the initially formed PS-rich block. In the DTHFP series, their frequency decreases with increasing DTHFP concentration, increasing *T*_g,S_. Similar behavior with increasing *T*_g,S_ (84–93 °C) was observed for the copolymers obtained using triglyme, 12C4, and 15C5 with increasing blockiness (87–96%). In good agreement with literature reports,^21^ the glass transition temperature of the isoprene-rich phase, *T*_g,I_, markedly increases from −41 °C up to 21 °C in the presence of chelating modifiers. This results from changes in PI regioisomeric composition: a decrease in 1,4-addition (from 96% to 25%) and increases in 1,2-addition (from 0.4% to 10%) and 3,4-addition (from 4% to 65%), which correlate with the thermal characteristics of polyisoprene (*T*_g,1,4‑PI_ = −66 °C; *T*_g,3,4‑PI_ = 33 °C; *T*_g,1,2‑PI_ = 9 °C).^52–56^ Compared to PI synthesized in pure THF, these copolymers have higher 1,4-PI and lower 1,2-PI content, but also a pronounced increase in 3,4-PI content, exceeding that of PI synthesized in pure THF.^21^ Interestingly, for the higher-coordinating modifiers (CN = 4–5), a preliminary analysis reveals relatively high 1,4-PI content (42–82%) alongside strongly accelerated styrene polymerization. Although similar behavior was reported for potassium counterions using potassium *tert*-amylate (KOAm), the explanation based on the HSAB concept on different active chain ends does not apply here.^29^ Instead, the steric effect of bulky modifiers (triglyme, 12C4, and 15C5) is proposed to stabilize the allylic chain end, thereby favoring 1,4-addition. It should be noted that, for the crown ethers the reported PI microstructure refers only to PI units incorporated within the PS-rich block, since incomplete polymerization prevented formation of a distinct PI end block.

In conclusion, the compositional gradient in anionic S/I copolymerization can be efficiently and systematically tuned by the electronic, steric, and coordination characteristics of ether-type polar modifiers. For monodentate ligands, their efficiency in modulating reactivity ratios correlates with their donor number. At constant ratio, [PM]/[Li] = 2, they lead to moderately flat gradients. In contrast, bi- and tridentate Lewis bases (DME, DTHFP, diglyme) enable access to inverse-tapered architectures (from *r*_I_ ≫ *r*_S_ to *r*_I_ ≪ *r*_S_) at constant overall composition, molar mass, and modifier-to-initiator ratio. *In situ* NIR kinetics further reveal that random copolymerization is achieved with only 0.25 equiv DTHFP, making it 20 times more efficient than THF. The high efficiency in chelating the lithium ion comes along with increased vinyl (1,2- and 3,4-) content of the PI-rich segments, strongly affecting thermal properties. Overall, bidentate DTHFP provides the most effective architectural control without loss of livingness, highlighting its potential for rational design of advanced thermoplastic elastomers. An increased vinyl content in PI enables postpolymerization modification, such as cross-linking or functionalization for various applications.

## Supplementary Material



## Notes

Comprehensive compilation of additional data, including materials, instrumentation, and experimental procedures; Polymer synthesis and characterization (SEC traces, NMR spectra, and DSC curves); Copolymerization kinetics and determination of reactivity ratios, blockiness, PI microstructure, and glass transition temperature; Molar- and volume-based composition diagrams and additional plots of each copolymer (PDF)
