# Supplementary material for: Shaping the Gradient: Ether-Type Polar Modifiers for the Statistical Anionic Copolymerization of Styrene and Isoprene
Source: ACS Macro Lett. 2026 May 28;15(6):878–84. doi: 10.1021/acsmacrolett.6c00201 (PMC13276885; doi:10.1021/acsmacrolett.6c00201)
Supplement: Supplementary file 1 [file mz6c00201_si_001.pdf]

Supporting Information

Shaping the Gradient: Ether-type Polar Modifiers for the Statistical Anionic Copolymerization of Styrene and Isoprene

Thi Dinh[1], Marvin Steube[1], Tobias Johann[1], Holger Frey[1*], and Axel H. E. Müller[1*]

[1]Department of Chemistry, Johannes Gutenberg-University Mainz, Duesbergweg 10-14, D-55128 Mainz, Germany

Table of Contents

1. Materials and Experimental Procedures 3

1.1. Chemicals 3

1.2. General Experimental Methods 3

1.3. Drying of Chemicals 3

2. Instrumentation 4

2.1. Near-Infrared Spectroscopy (NIR Spectroscopy) 4

2.2. Temperature Logger 4

2.3. Size Exclusion Chromatography (SEC) 4

2.4. Nuclear Magnetic Resonance Spectroscopy (NMR) 5

2.5. Differential Scanning Calorimetry (DSC) 5

3. Polymer Synthesis and Characterization 6

3.1. Synthesis of P(I-*co*-S) Copolymers 6

3.2. Additional Polar Modifier Series 7

3.3. Molar Mass Distribution by SEC 9

3.4. NMR Spectra 12

4. Copolymerization Kinetics and Determination of Reactivity Ratios 25

4.1. Temperature Profiles 25

4.2. *In Situ* NIR Monitoring 28

4.3. Time-Dependent Individual Concentration Plots ... 29

4.4. Individual vs Total Conversion Plots ... 31

4.5. Fitting Method ... 33

5. Investigation by NMR and DSC ... 35

5.1. Determination of Blockiness of Styrene Units *via* NMR ... 35

5.2. Determination of PI Microstructure *via* NMR ... 37

5.3. Determination of Thermal Properties *via* DSC ... 40

6. Visualization of Experimental Results ... 43

6.1. Copolymer Composition Diagrams ... 43

6.2. Plots of Individual Time-Conversion, Individual *vs* Total Conversion, and Reactivity Ratios for THT Series as well as Plots of (Nomalized) Half-lives ... 45

6.3. Relation of Reactivity Ratios and Donor Number for Monodentate Polar Modifiers... 46

6.4. Plots of Glass Transition Temperature, Blockiness, and PI Microstructure for Additional Polar Modifier Series ... 47

7. References ... 48

1. Materials and Experimental Procedures

1.1. Chemicals

All chemicals and solvents were purchased from *Sigma Aldrich*, *Fischer Scientific*, *Roth*, *TCI*, and *abcr*, whereas deuterated solvents were obtained from the company *Deutero GmbH*. While the initiator and chemicals used for the drying of solvents and polar additives were used without further purification, all solvents, monomers, and polar modifiers were freed from the stabilizer, dried, and freshly distilled prior to use.

1.2. General Experimental Methods

When required, all reactions were carried out using standard Schlenk techniques. A constant pressure of 50 mbar was maintained by installing a pressure reducer between the argon cylinder and the Schlenk line.

All glassware and reaction vessels were flamed-dried under high vacuum (10^{-3} mbar) three times prior to use, and subsequently filled with dry argon to establish an inert gas atmosphere.

During cryo-transfer processes of monomer and polar modifiers, the receiving flask or ampoule was cooled with liquid nitrogen. Transfers were conducted either at room temperature or, if required, at elevated temperature by heating the distillation flask in a water bath. All transfer processes were performed under static high vacuum (10^{-3} mbar).

1.3. Drying of Chemicals

High-purity argon (5.0) was purified prior to use by passage through multiple drying solutions containing cyclohexane (CyH), *sec*-butyllithium (*s*-BuLi), and diphenylethylene (DPE) and was subsequently supplied via a Schlenk line.

Prior to use, stabilizers were removed from isoprene and styrene by filtration through a chromatography column packed with basic aluminum oxide. The monomer mixture was then dried sequentially over calcium hydride (CaH_2) for 1 day at room temperature, followed by drying over trioctylaluminum ($Al(Oct)_3$) for an additional day at room temperature.

Cyclohexane (CyH), used as the polymerization solvent, was dried by distillation over sodium and benzophenone under reflux in an argon atmosphere for at least 4 days.

The polar modifiers were either dried twice over CaH_2 or over diphenylethylenlithium, generated *in situ* from *s*-BuLi and a slight excess of DPE (1:1.2 or 1:1.05) at room temperature to prevent side reactions.

2. Instrumentation

2.1. Near-Infrared Spectroscopy (NIR Spectroscopy)

NIR spectra were measured on a *Nicolet Magna 560* FT-IR spectrometer with a PbS detector and a CaF_2 beam splitter using the *Omnic 7.4* software from the company *Thermo Scientific*. The NIR probe was connected to the detector *via* glass fibers, and an IR laser source served as the light source, with an aperture of 88, a mirror speed of 0.6329, and an internal ADC amplification of 8. All spectra were recorded in the range of 5900 cm^{-3} to 6250 cm^{-3}.

The obtained NIR spectra were plotted, processed, and evaluated using the *NIREVAL V3* software, developed by Dr. Tobias Johann. Deconvolution was performed analogously to one of our previous work[1].

2.2. Temperature Logger

Temperature profiles were recorded at 10 s intervals with a *LOG200-E* temperature logger, which was attached to an external temperature sensor. The calibration was certified by *Dostmann electronic GmbH* (Germany) (T = 23 °C, Deviation = $\pm$ 0.5 K; Uncertainty = $\pm$ 0.5 K).

2.3. Size Exclusion Chromatography (SEC)

Size Exclusion Chromatography (SEC) was performed using an *Agilent 1260 Infinity II* setup equipped with an MZ-Gel SD*plus* 10^5/10^3/100 Å column set from the company *MZ-Analysetechnik* (Mainz, Germany). The detection of polymers was achieved using a refractive index (RI) detector (Agilent G1362A) and a UV detector (TSP UV 254 nm).

Tetrahydrofuran (THF) served as the eluent at a flow rate of 1 mL min^{-1}, with an injection volume of 100 µL, and all measurements were conducted at 30 °C. Molecular weights were determined by calibration with polystyrene (PS) standards purchased from *PSS Polymer Standard Service GmbH* (Mainz, Germany), using toluene as an internal standard.

Data acquisition and evaluation were performed using the software PSS *WinGPC UniChrom V.8.31, Build 8417*, developed by *PSS Polymer Standard Service GmbH* (Mainz, Germany). The SEC software,

developed by Dr. J. Blankenburg, was used to plot the SEC traces. Unless stated otherwise, all SEC chromatograms display the RI-detector signal.

For sample preparation, approximately 1 mg of polymer was dissolved in 1.5 mL of THF, followed by the addition of one drop of toluene.

2.4. Nuclear Magnetic Resonance Spectroscopy (NMR)

The NMR spectra were recorded on a *Bruker Avance III 600* spectrometer at 600 MHz for ^{1}H-NMR and 150 MHz for *inverse-gated* ^{13}C-NMR. Chemical shifts (δ) are reported in parts per million (ppm) and referenced to the signal of protons within the deuterated solvent. All measurements were performed in deuterated chloroform ($CDCl_3$).

Spectral evaluation was conducted using the software *MestReNova v14.3.3* developed by *Mesrelab Research S. L.* (Santiago de Compostela, Spain). The signals of protons were labeled with small letters, while the corresponding carbon signals were denoted by uppercase letters. Identical letters were used to assign protons and carbons belonging to the same structural unit, ensuring consistent signal assignment.

Samples were prepared by dissolving approximately 100 mg of polymer in 7 mL of $CDCl_3$.

2.5. Differential Scanning Calorimetry (DSC)

The heating curves were measured with a *TA Instruments DSC 250* connected to a *TA Instruments RCS 90* refrigeration system from *Waters Corporation* (United States), and the data were analyzed using the *TRIOS* software by *Waters Corporation* (United States).

Two heating and one cooling cycle were performed at a heating/cooling rate of 20 K/min with a two-point calibration of *n*-octane and indium in a temperature range between –90 °C and 150 °C. The glass transition temperatures were then extracted from the second heating cycle.

Samples were prepared by weighing at least 5 mg of dried polymer into a pan, which was subsequently sealed.

3. Polymer Synthesis and Characterization

3.1. Synthesis of P(I-*co*-S) Copolymers

The synthesis was adapted from the literature of Steube *et al.*.[1]

Example:

Synthesis of the equimolar 80 kg mol^{-1} copolymer P(I-*co*-S) in cyclohexane (CyH) with the addition of 2 eq DTHFP.

All syntheses of the remaining copolymers in pure CyH in the absence and the presence of the other polar modifiers, with constant or increasing modifier content, were carried out in the same way.

Procedure:

The gradient copolymer was synthesized in a 1 L anionic flask. To 700 mL of dry cyclohexane (CyH), 1.0 mL of a dry DTHFP stock solution (0.43 mL, 2.15 mmol, 2 eq) was added as a polar modifier. The solution was temperature-controlled at 20° C using a water bath connected to an external thermostat. Temperature measurements were conducted in this water bath in direct contact with the reaction vessel. Upon reaching the temperature of 20 °C, the *in situ* NIR kinetic measurement was started. Subsequently, 117 mL of a dried equimolar monomer mixture consisting of styrene (0.54 mol, 464 eq) and isoprene (0.54 mol, 464 eq) was added via a graduated ampoule under stirring. The polymerization was initiated with 0.9 mL (1.17 mmol, 1 eq) of 1.3 M *s*-BuLi initiator solution.

After completion of the polymerization, the reaction was terminated by the addition of 1 mL methanol (MeOH) and was stirred for an additional 5 minutes until complete decolorization. The polymer was isolated by precipitation into a 5-fold volume excess of a mixture of isopropyl alcohol (*i*PrOH) and MeOH (volume ratio of 1:4), followed by two washing steps with *i*PrOH. The recovered polymer was dried under vacuum for at least 1 week at room temperature stored at –20° C. The pure, colorless polymer P(I-*co*-S) was obtained in a quantitative yield.

3.2. Additional Polar Modifier Series

Table S1: Experimental conditions and results of the copolymerization of styrene and isoprene in cyclohexane and in the presence of various polar modifiers, PM.[a]

Polar Modifier	[PM]/[Li]	M_n[b] [kg mol^{-1}]	Đ[b]	x_I[c] [%]	$t_{1/2,I}$[d] [min]	$t_{1/2,S}$[d] [min]	r_I[e]	r_S[e]	$r_I \cdot r_S$[e]	B[f] [%]	1,4-/3,4-/1,2-PI[f] [%]	T_g[g] [°C]	Molar Gradient[h]
**THT**	0	91.4	1.09	100	118	680	10.4	0.007	0.073	74	96/4/0.4	−41/102	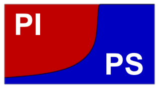
**THT**	2	71.6	1.11	100	126	722	8.69	0.009	0.080	74	95/5/0.2	−41/99	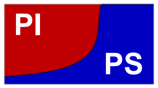
**THT**	20	77.1	1.08	100	63	335	8.92	0.010	0.089	73	95/5/0.3	−36/101	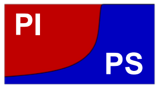
**–**	–	91.4	1.09	100	118	680	10.4	0.007	0.073	74	96/4/0.4	−41/102	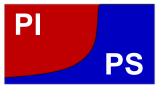
**DME**	2	70.7	1.09	100	16	2.6	0.438	5.77	2.53	81	34/59/6.7	17/75	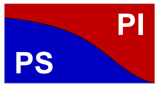
**Diglyme**	2	74.8	1.32	>98	36	3.1	0.369	10.5	3.88	85	31/60/8.6	16/80	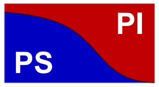
**Triglyme**	2	59.3	1.45	~46	n.a.	n.a.	n.a.	n.a.	n.a.	(87)[i]	(42/44/14)[i]	(n.a./84)[i]	n.a

Table S1 *(continued)*

Polar Modifier	[PM]/[Li]	M_n[b] [kg mol^{-1}]	$Đ$[b]	x_I[c] [%]	$t_{1/2,I}$[d] [min]	$t_{1/2,S}$[d] [min]	r_I[e]	r_S[e]	$r_I \cdot r_S$[e]	B[f] [%]	1,4-/3,4-/1,2-PI[f] [%]	T_g[g] [°C]	Gradient[h]
**–**	–	91.4	1.09	100	118	680	10.4	0.007	0.073	74	96/4/0.4	–41/102	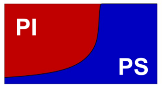
**12C4**	2	53.6	1.95	~7	n.a	n.a	n.a	n.a	n.a	(96)[i]	(82/16/3.6)[i]	(n.a./93)[i]	n.a
**15C5**	2	89.6	1.36	~10	n.a	n.a	n.a	n.a	n.a	(94)[i]	(72/21/6.7)[i]	(n.a./91)[i]	n.a

[a]Equimolar monomer feed of $50\%_{mol} = 60\%_{wt} = 57\%_{vol}$ styrene, $[S]_0 = [I]_0 = 0.65$ mol L^{-1}, [*s*-BuLi]$_0$ = 1.44 mmol L^{-1}, T = ~20 °C. [b]Isoprene conversion determined from ^{1}H-NMR spectroscopy [c]Molar masses, M_n, and dispersities, $Đ$, determined *via* SEC (THF, PS-standards, RI-detector).[d]Half-lives, $t_{1/2,I}$ and $t_{1/2,S}$, determined from time-conversion plots, [e]Reactivity ratios, r_I, r_S and $r_I \cdot r_S$, determined from Meyer-Lowry fits, [f]Blockiness, B, defined as the molar fraction of at least two consecutive (block-like) styrene units and content of polyisoprene microstructure, 1,4-/3,4-/1,2-PI, determined from ^{1}H- and *inverse-gated* ^{13}C-NMR spectroscopy (see **Chapter 5** and **Figures S7–S21**), [g]Glass transition temperatures determined from DSC using the second heating curve (**Figures S41–S45**). [h]Molar-based composition diagram as a plot of instantaneous styrene incorporation, F_S, *vs* total monomer conversion to visualize copolymer composition. [i]Estimated data due to incomplete reactions.

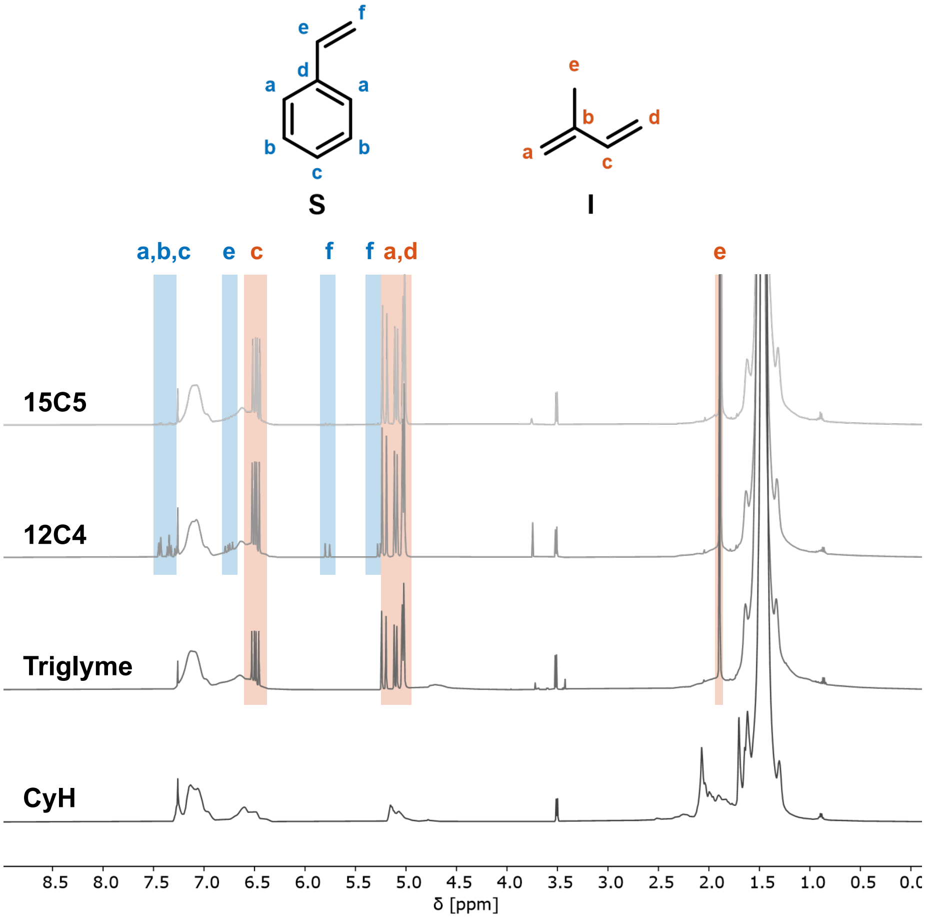

Figure S1: Crude ^{1}H-NMR spectra (400 MHz, $CDCl_3$) of terminated S/I copolymerizations in pure CyH (complete reaction) and in the presence of diglyme, triglyme, 12C4, and 15C5 (incomplete reaction), with marked signals of unreacted isoprene (orange) and styrene (blue).

3.3. Molar Mass Distribution by SEC

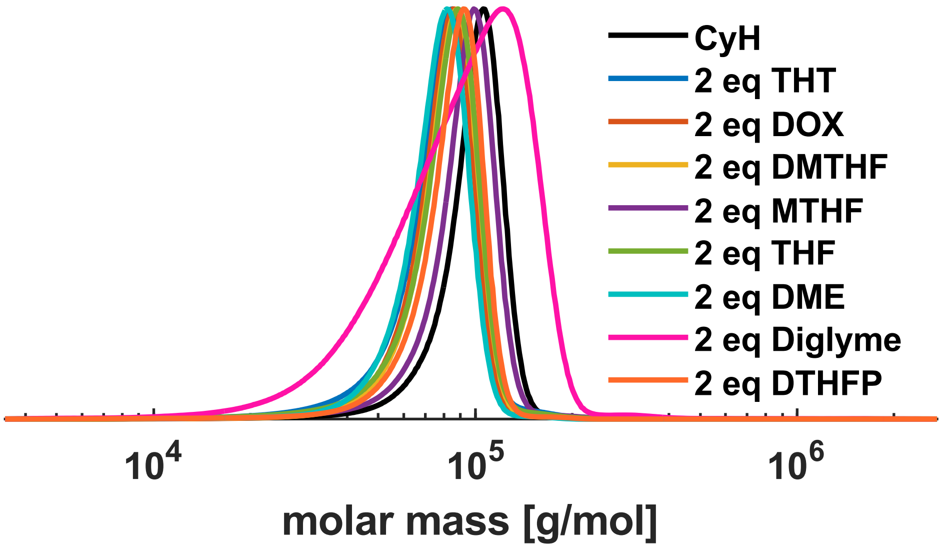

Figure S2: SEC traces of P(I-*co*-S) copolymers for [PM]/[Li] = 2, obtained using THF as eluent, PS-standards and the RI-detector.

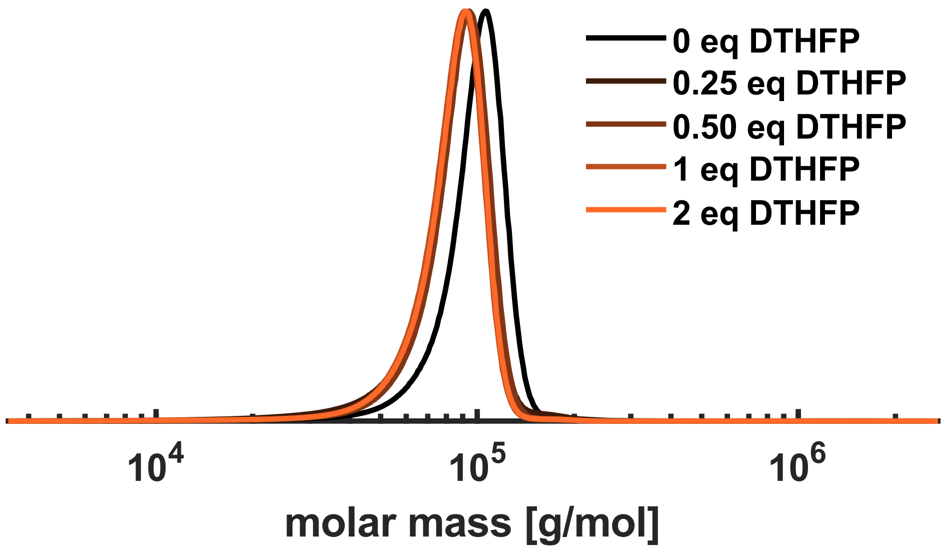

Figure S3: SEC traces of P(I-*co*-S) copolymers for various [DTHFP]/[Li] ratios, obtained using THF as eluent, PS standards, and the RI detector.

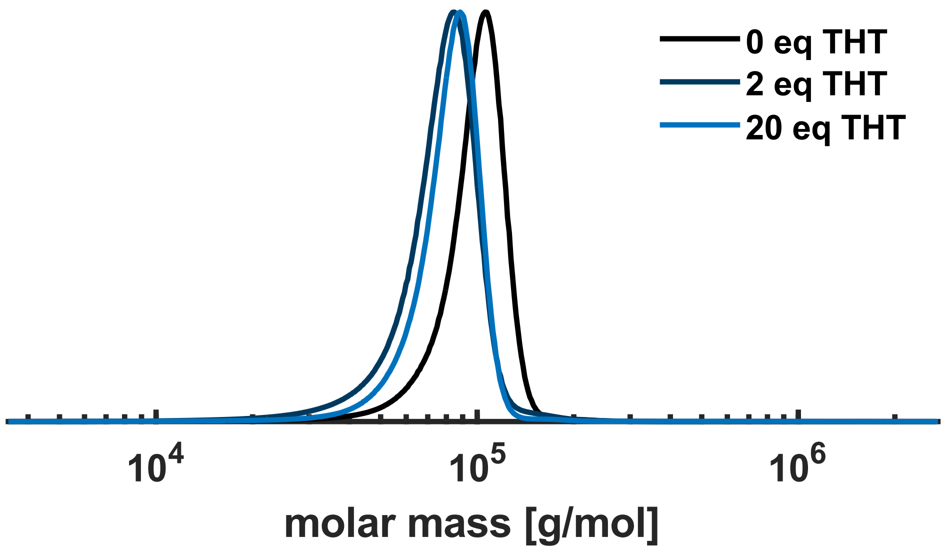

Figure S4: SEC traces of P(I-*co*-S) copolymers for various [THT]/[Li] ratios, obtained using THF as eluent, PS standards, and the RI detector.

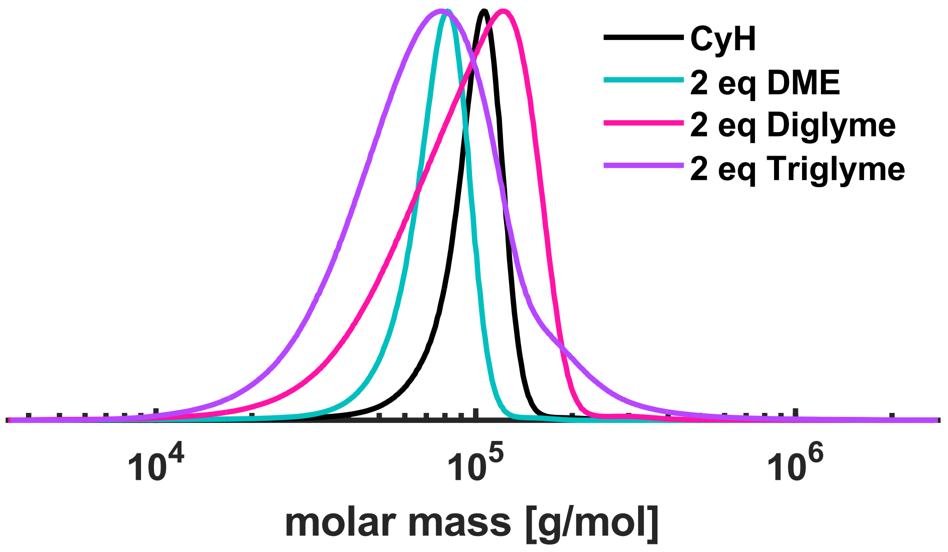

Figure S5: SEC traces of P(I-*co*-S) copolymers for [Glyme]/[Li] = 2, obtained using THF as eluent, PS-standards and the RI-detector.

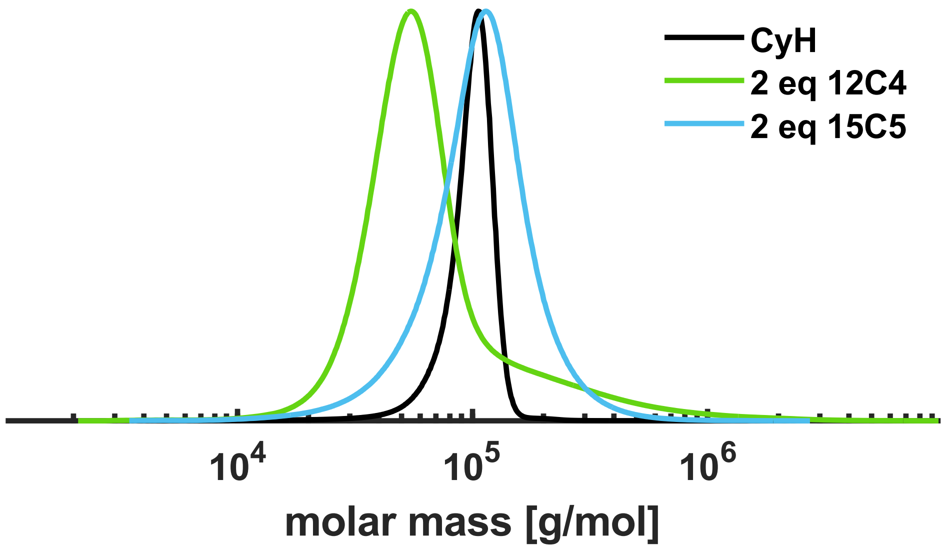

Figure S6: SEC traces of P(I-*co*-S) copolymers for [Crown Ether]/[Li] = 2, obtained using THF as eluent, PS-standards and the RI-detector.

3.4. NMR Spectra

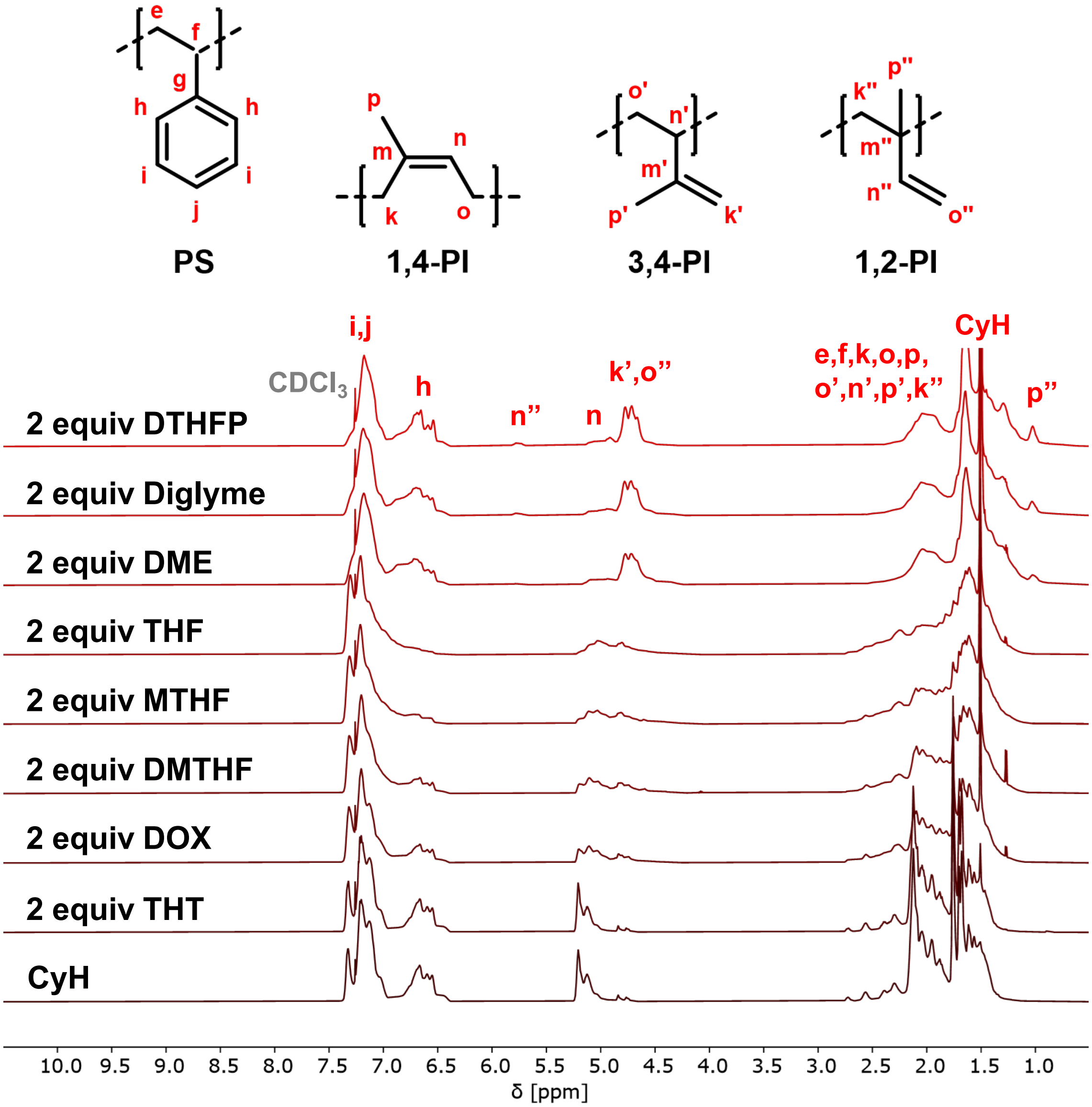

Figure S7: Stacked ^{1}H-NMR spectra (600 MHz, $CDCl_3$) of P(I-*co*-S) copolymers for [PM]/[Li] = 2.

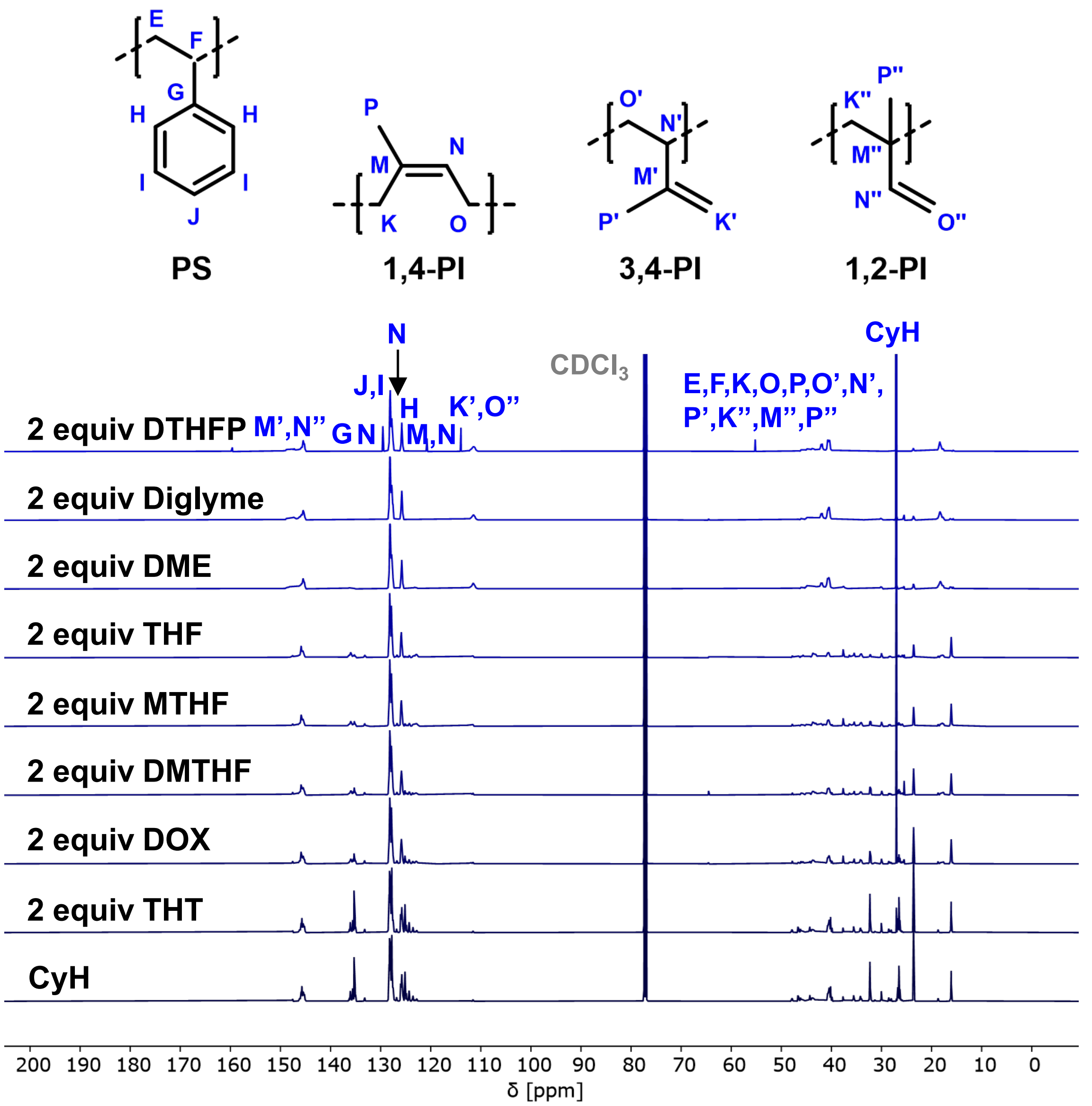

Figure S8: Stacked *inverse-gated* ^{13}C-NMR spectra (150 MHz, $CDCl_3$) of P(**I**-*co*-S) copolymers for [PM]/[Li] = 2.

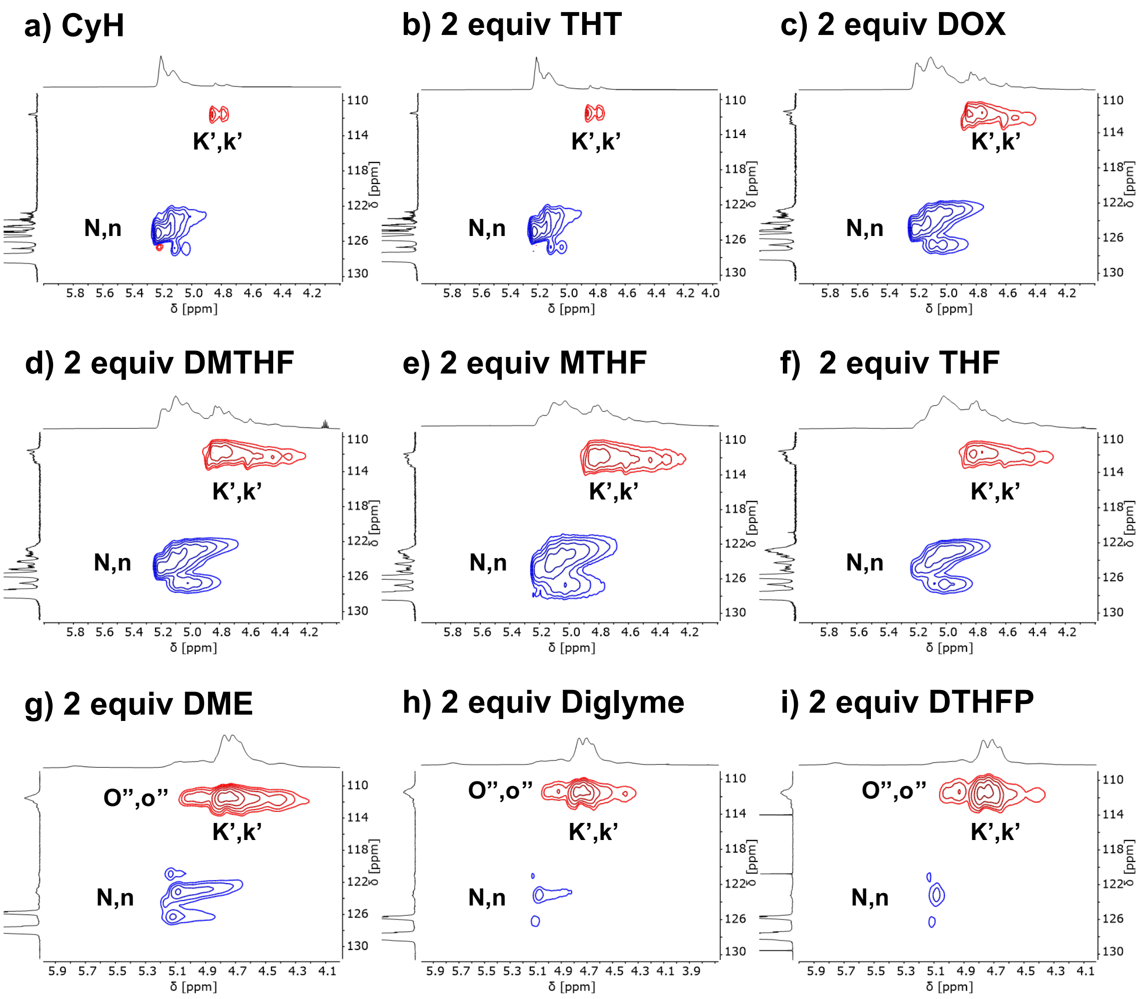

Figure S9: HSQC NMR spectra ($CDCl_3$) of P(I-*co*-S) copolymers for [PM]/[Li] = 2.

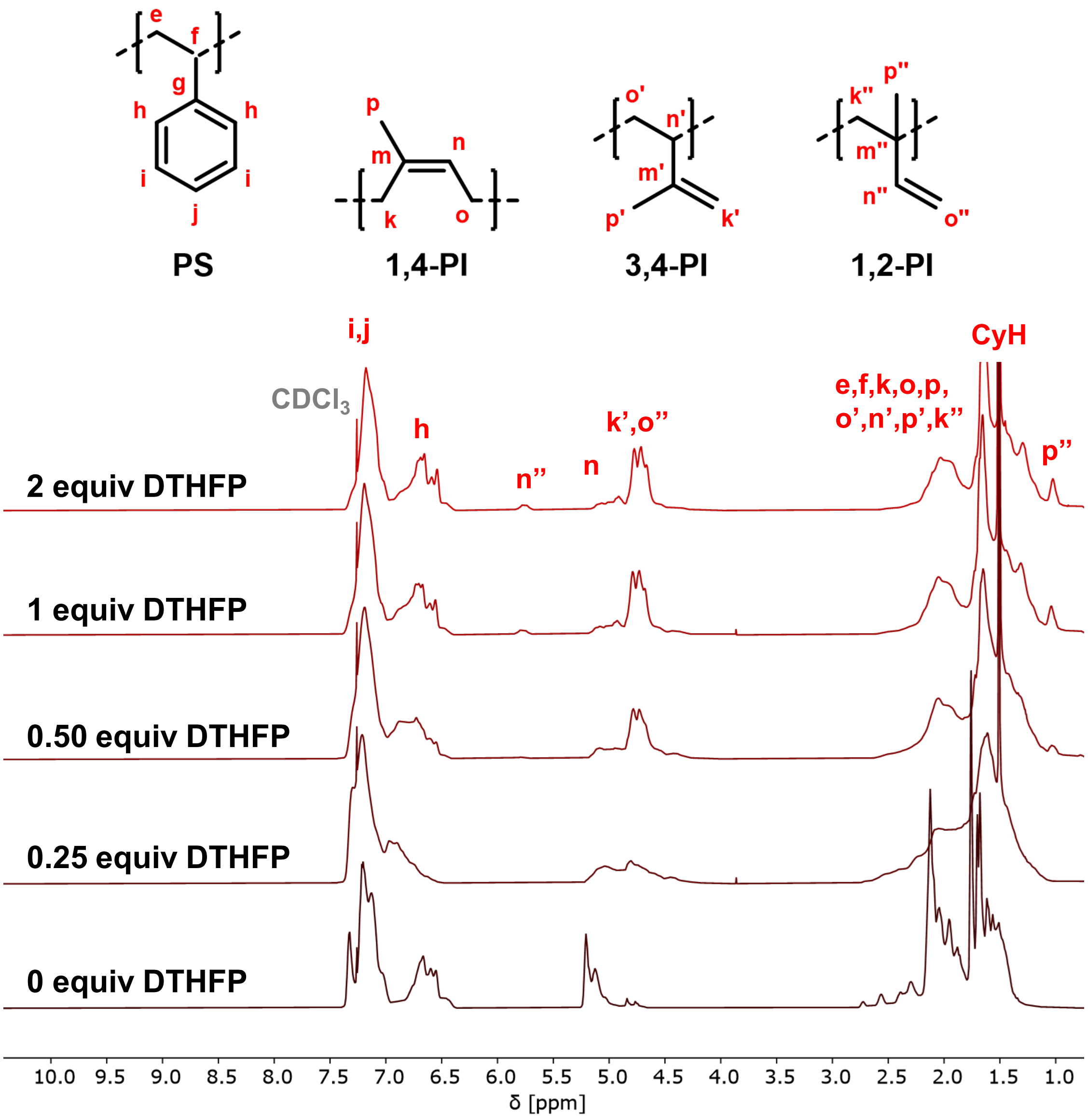

Figure S10: Stacked ^{1}H-NMR spectra (600 MHz, $CDCl_3$) of P(I-*co*-S) copolymers for various [DTHFP]/[Li] ratios.

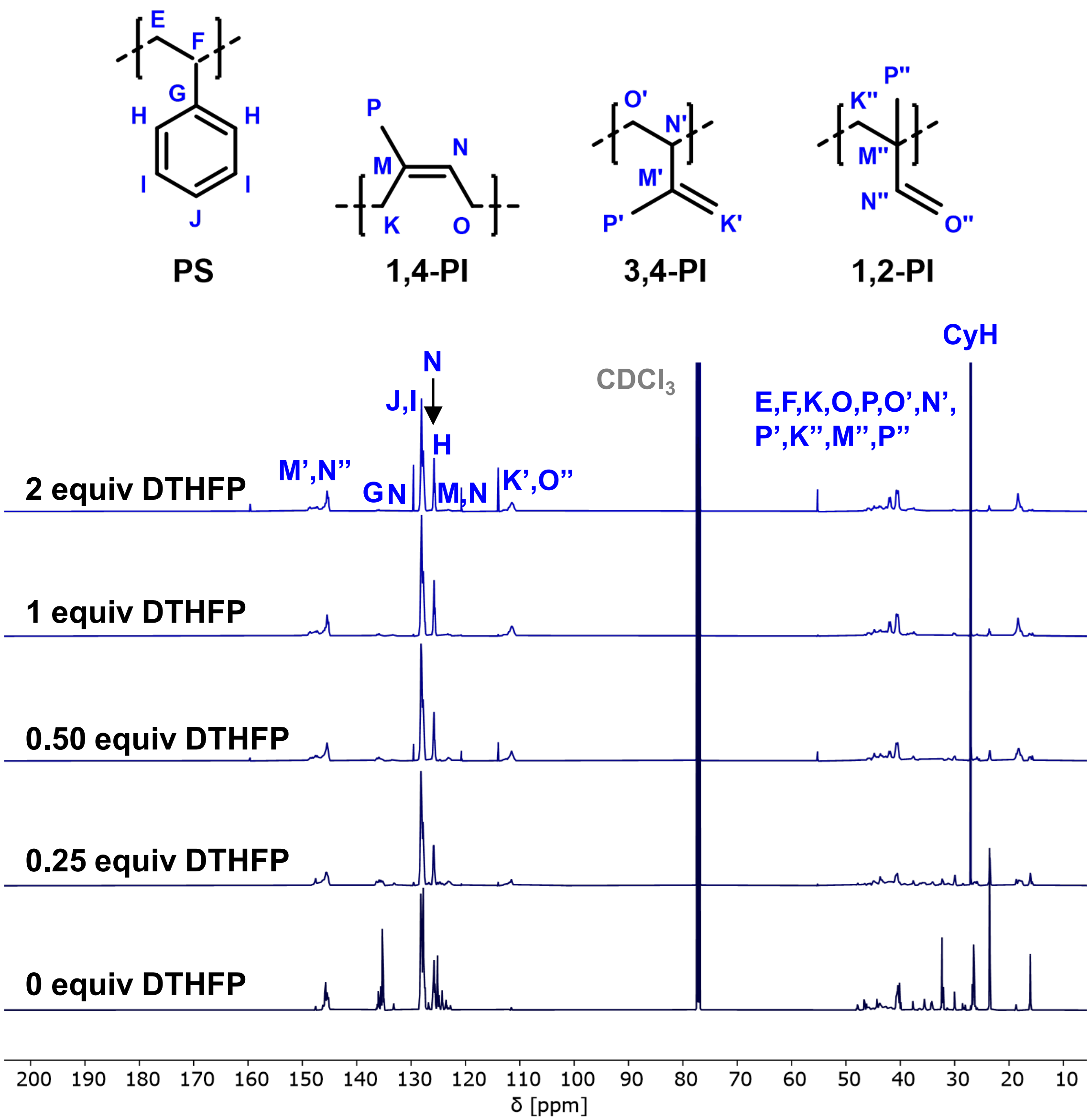

Figure S11: Stacked *inverse-gated* ^{13}C-NMR spectra (150 MHz, $CDCl_3$) of P(I-*co*-S) copolymers for various [DTHFP]/[Li] ratios.

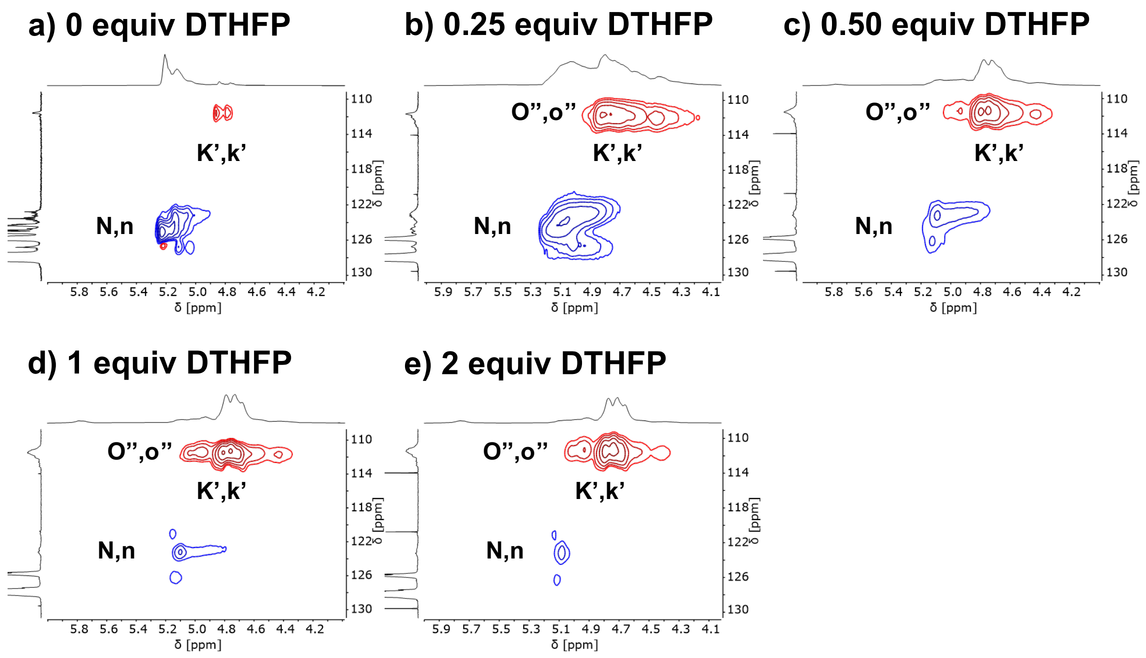

Figure S12: HSQC NMR spectra ($CDCl_3$) of P(I-*co*-S) copolymers for various [DTHFP]/[Li] ratios.

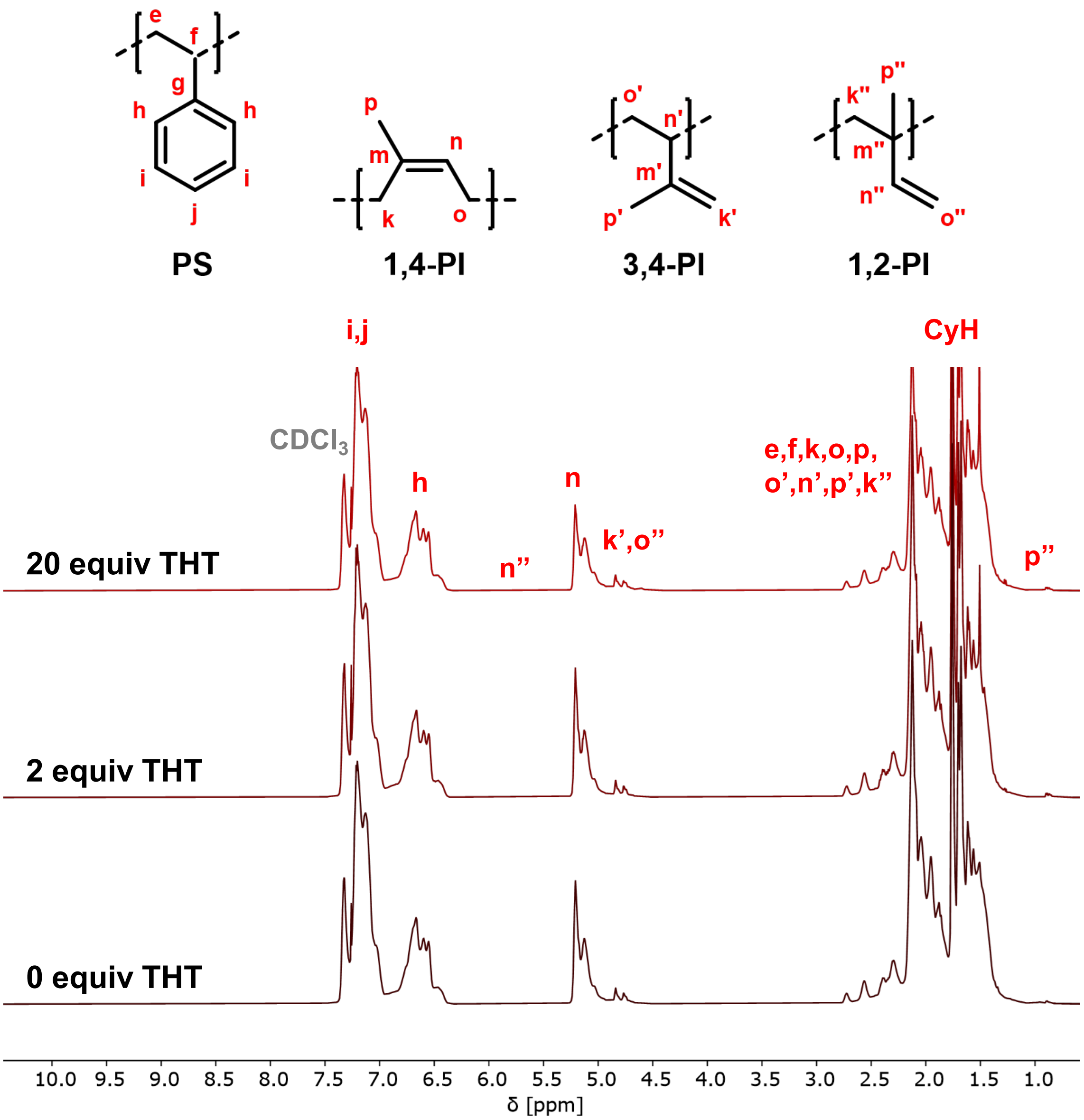

Figure S13: Stacked ^{1}H-NMR spectra (600 MHz, $CDCl_3$) of P(I-*co*-S) copolymers for various [THT]/[Li] ratios.

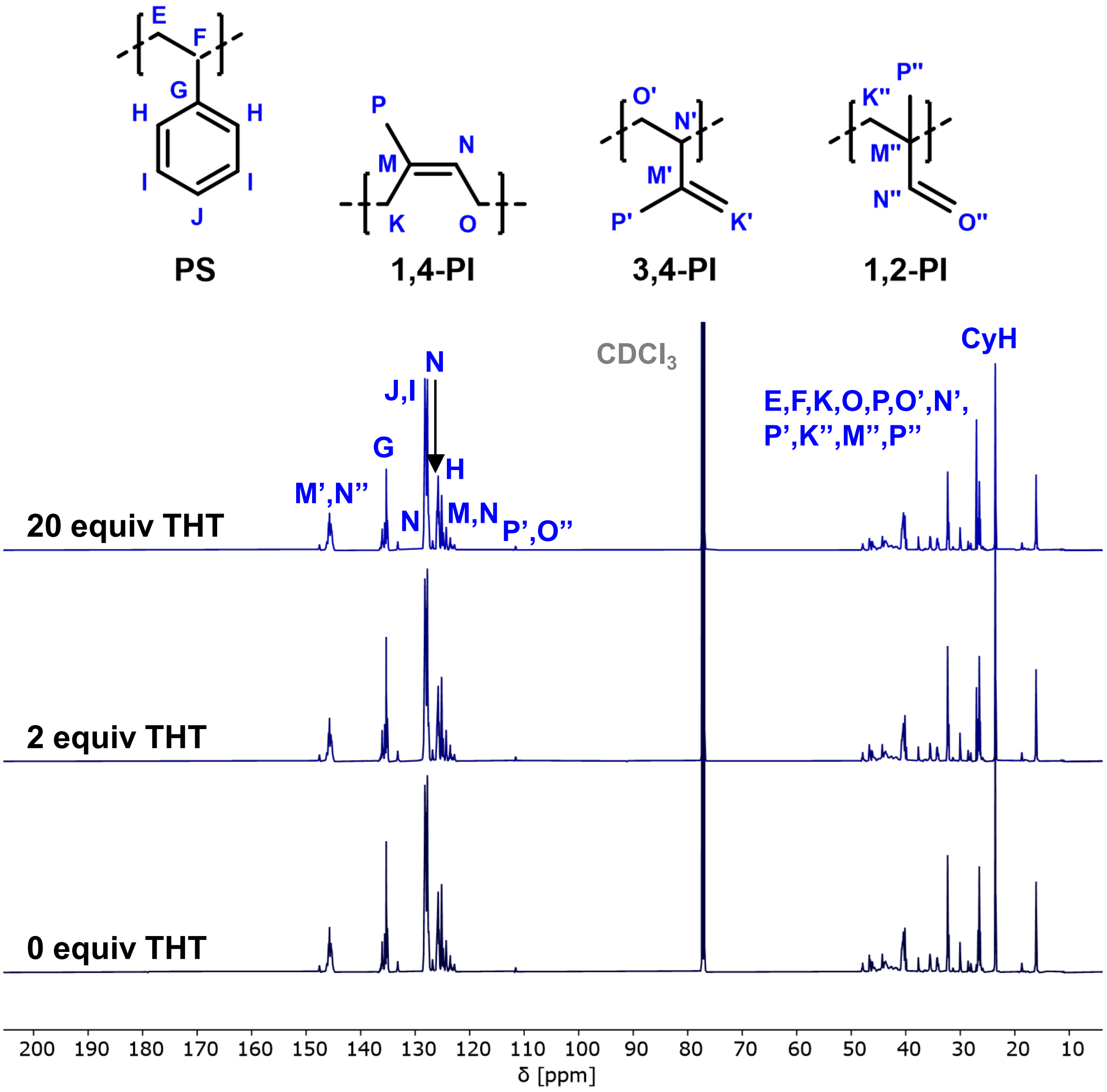

Figure S14: Stacked *inverse-gated* ^{13}C-NMR spectra (150 MHz, $CDCl_3$) of P(I-*co*-S) copolymers for various [THT]/[Li] ratios.

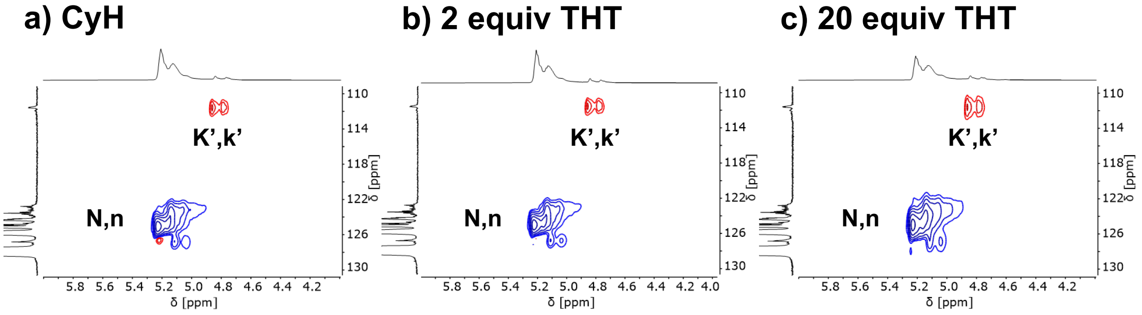

Figure S15: HSQC NMR spectra ($CDCl_3$) of P(I-*co*-S) copolymers for various [THT]/[Li] ratios.

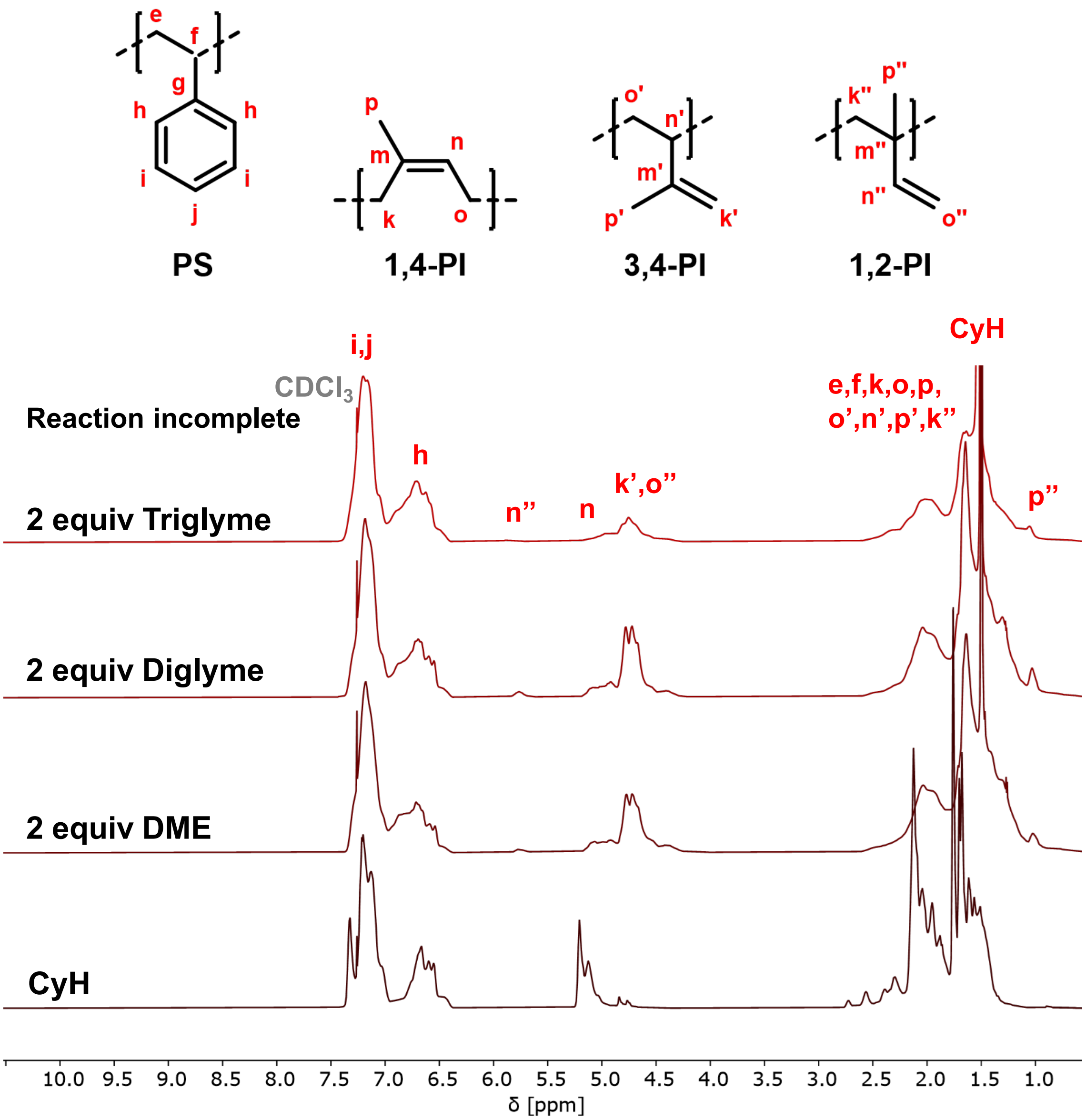

Figure S16: Stacked ^{1}H-NMR spectra (600 MHz, $CDCl_3$) of P(I-*co*-S) copolymers for [Glyme]/[Li] = 2.

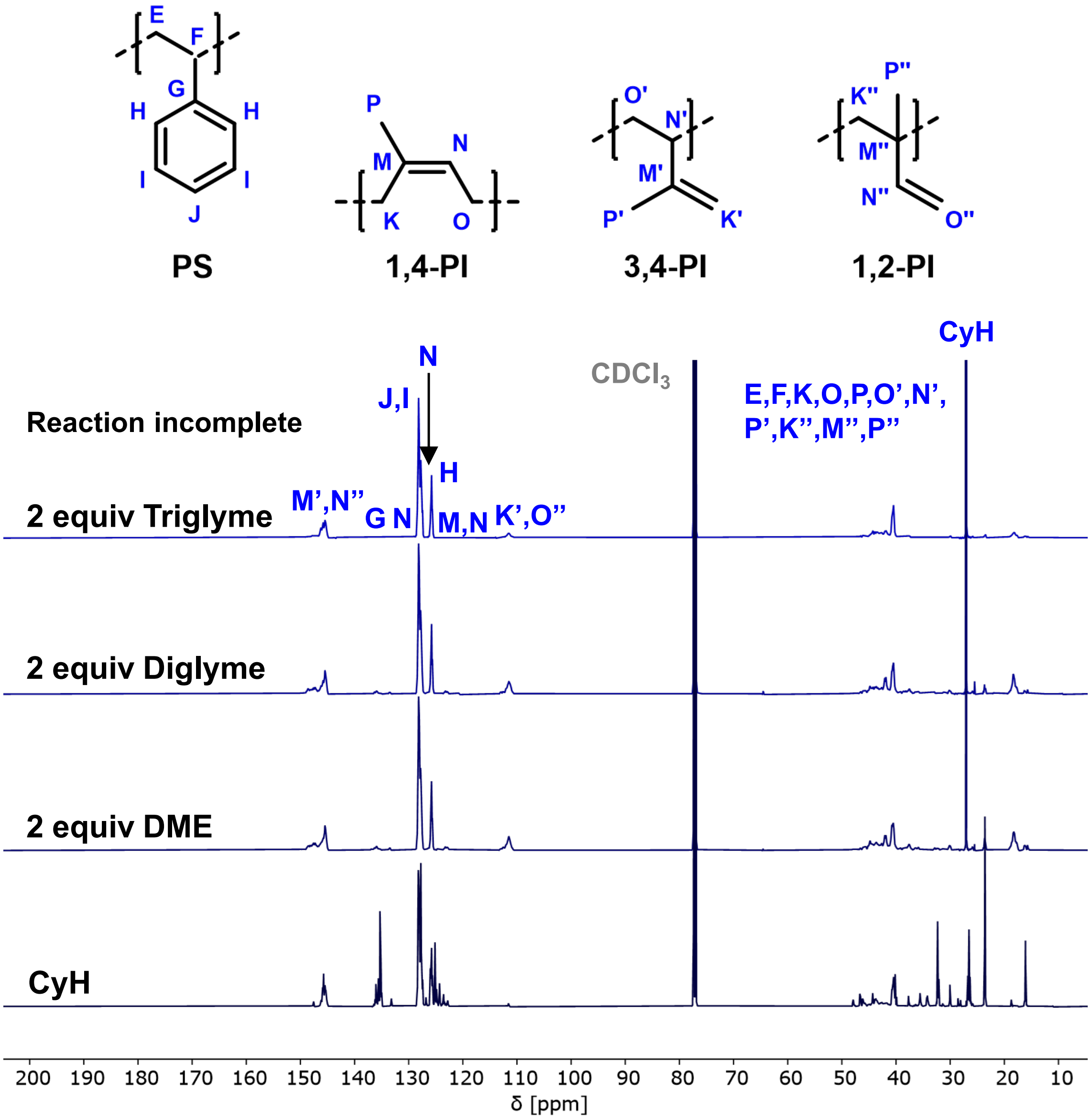

Figure S17: Stacked *inverse-gated* ^{13}C-NMR spectra (150 MHz, $CDCl_3$) of P(I-*co*-S) copolymers for [Glyme]/[Li] = 2.

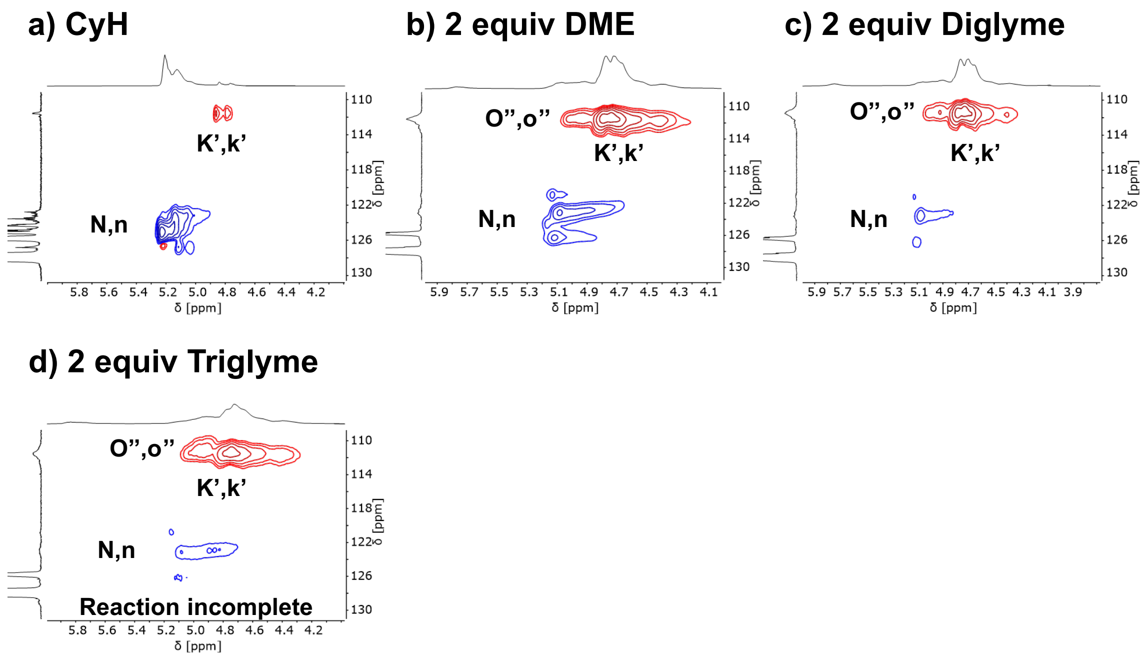

Figure S18: HSQC NMR spectra ($CDCl_3$) of P(I-*co*-S) copolymers for [Glyme]/[Li] = 2.

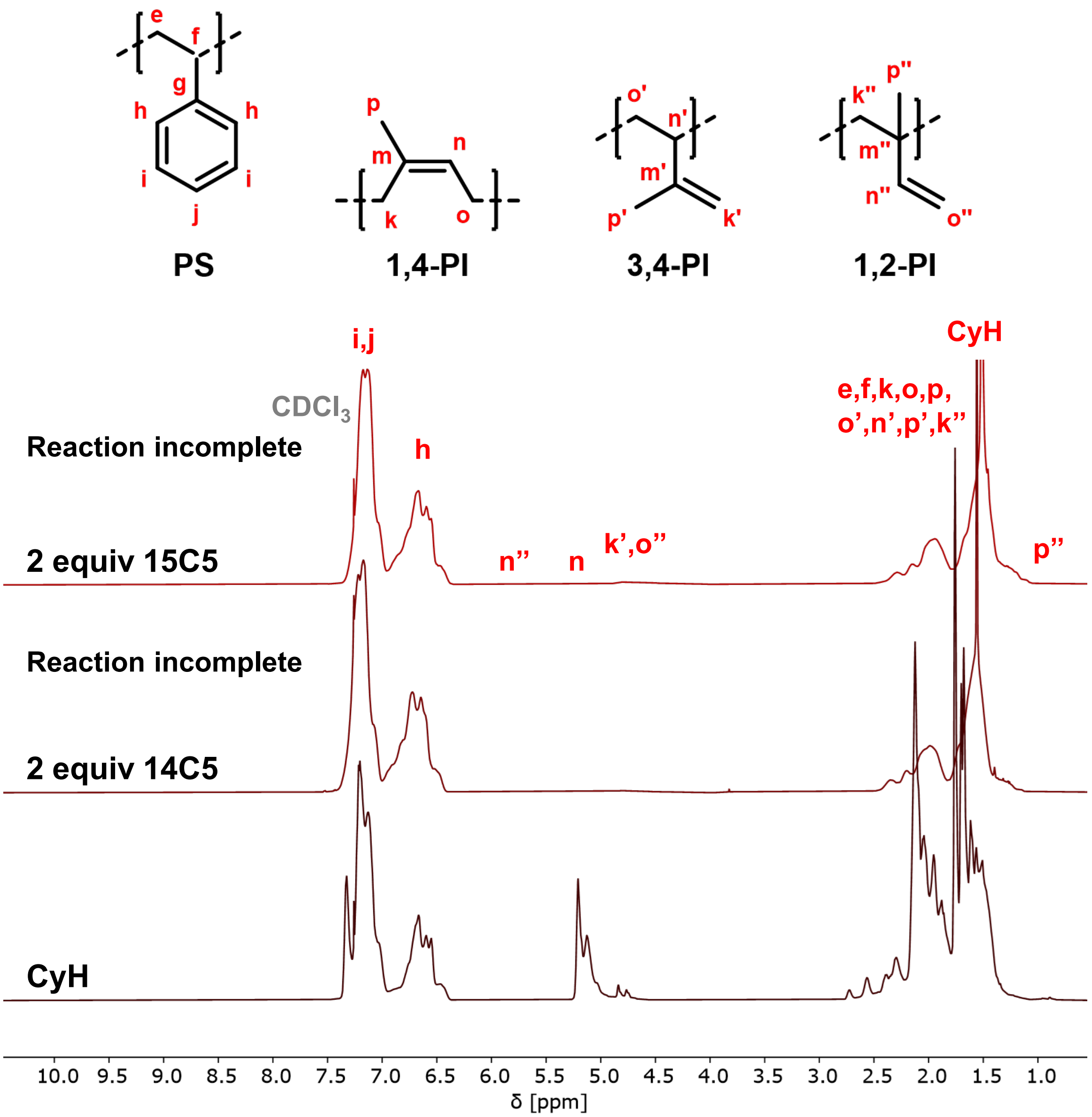

Figure S19: Stacked ^{1}H-NMR spectra (600 MHz, $CDCl_3$) of P(I-*co*-S) copolymers for [Crown Ether]/[Li] = 2.

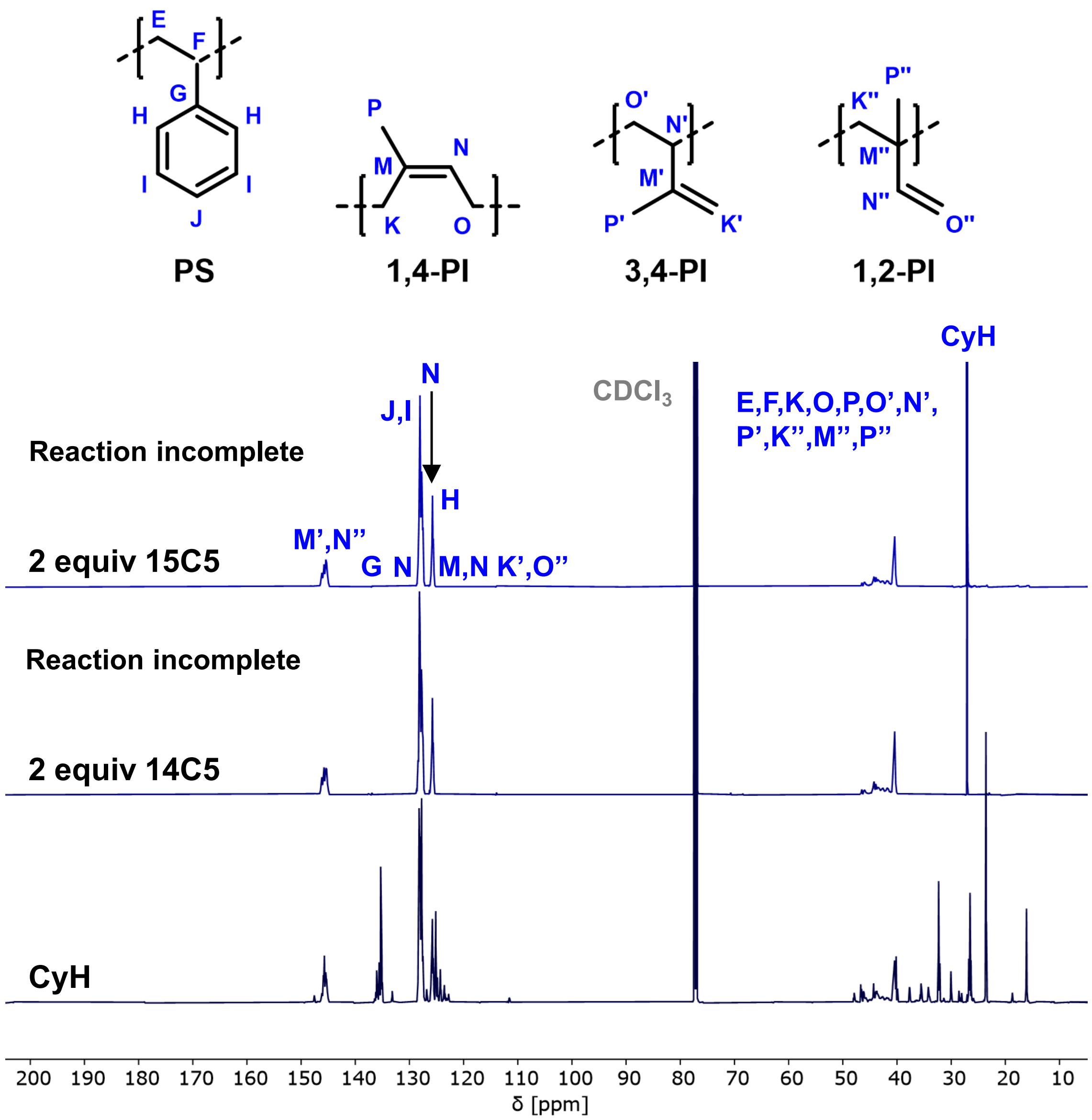

Figure S20: Stacked *inverse-gated* ^{13}C-NMR spectra (150 MHz, $CDCl_3$) of P(I-*co*-S) copolymers for [Crown Ether]/[Li] = 2.

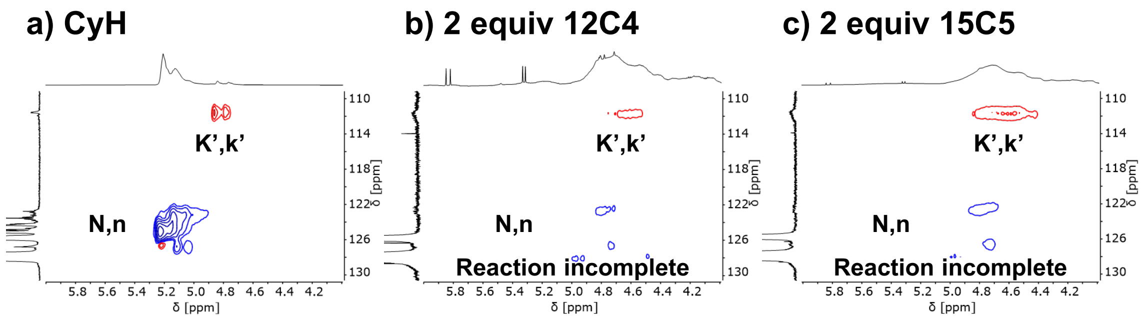

Figure S21: HSQC NMR spectra ($CDCl_3$) of P(I-*co*-S) copolymers for [Crown Ether]/[Li] = 2.

4. Copolymerization Kinetics and Determination of Reactivity Ratios

4.1. Temperature Profiles

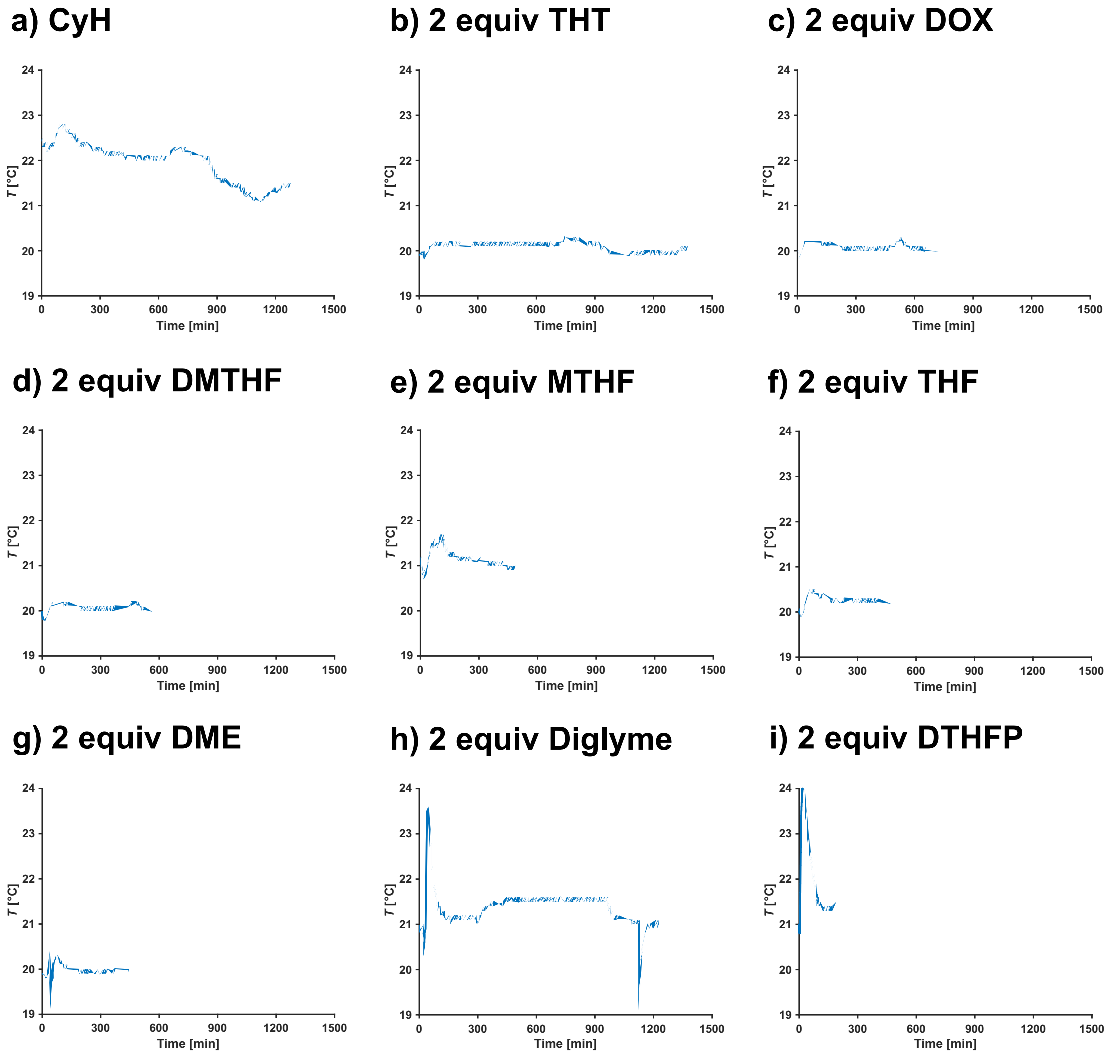

Figure S22: Temperature profiles during S/I copolymerization recorded outside the reaction vessel for [PM]/[Li] = 2.

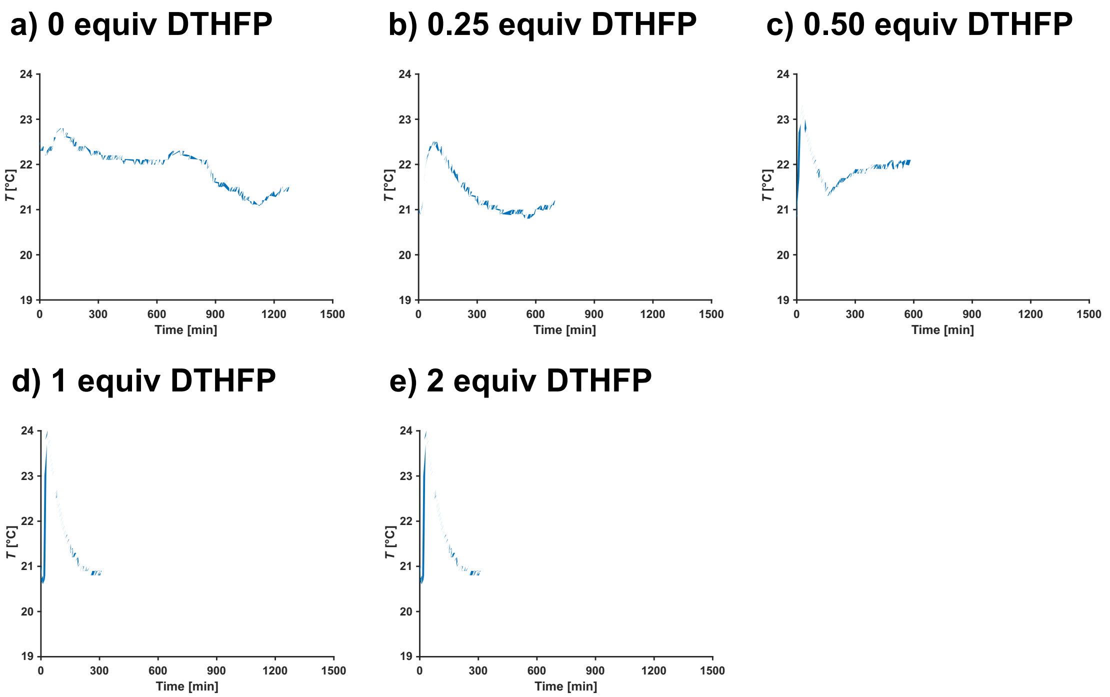

Figure S23: Temperature profiles during S/I copolymerization recorded outside the reaction vessel for various [DTHFP]/[Li] ratios.

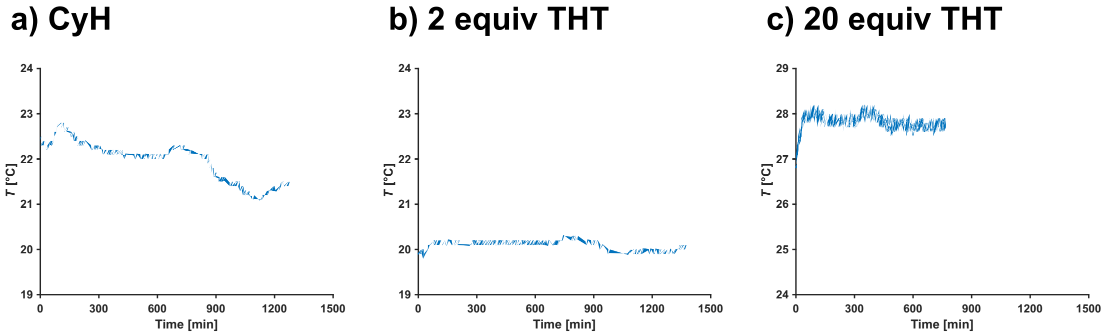

Figure S24: Temperature profiles during S/I copolymerization recorded outside the reaction vessel for various [THT]/[Li] ratios.

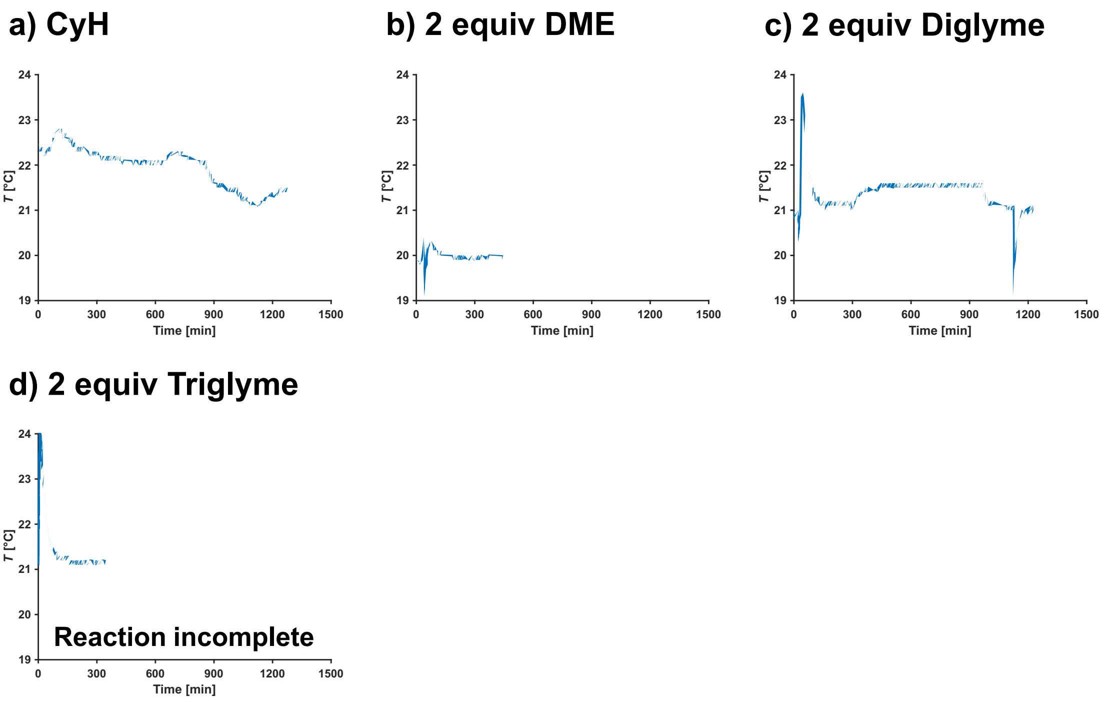

Figure S25: Temperature profiles during S/I copolymerization recorded outside the reaction vessel for [Glyme]/[Li] = 2.

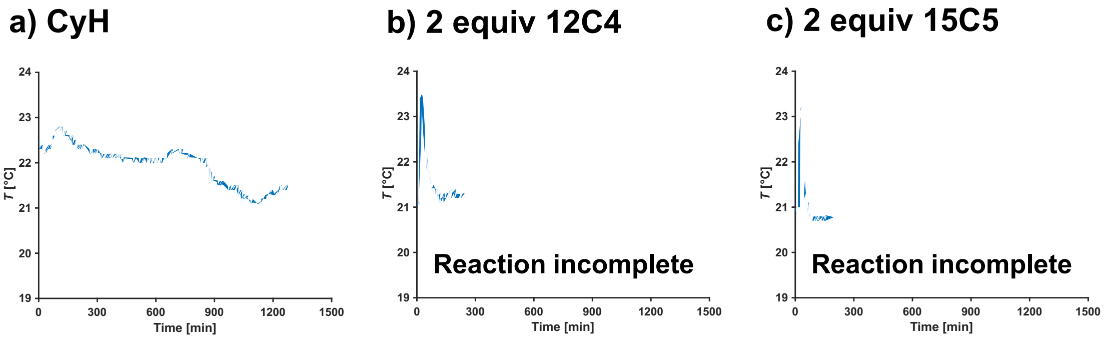

Figure S26: Temperature profiles during S/I copolymerization recorded outside the reaction vessel for [Crown Ether]/[Li] = 2.

4.2. *In Situ* NIR Monitoring

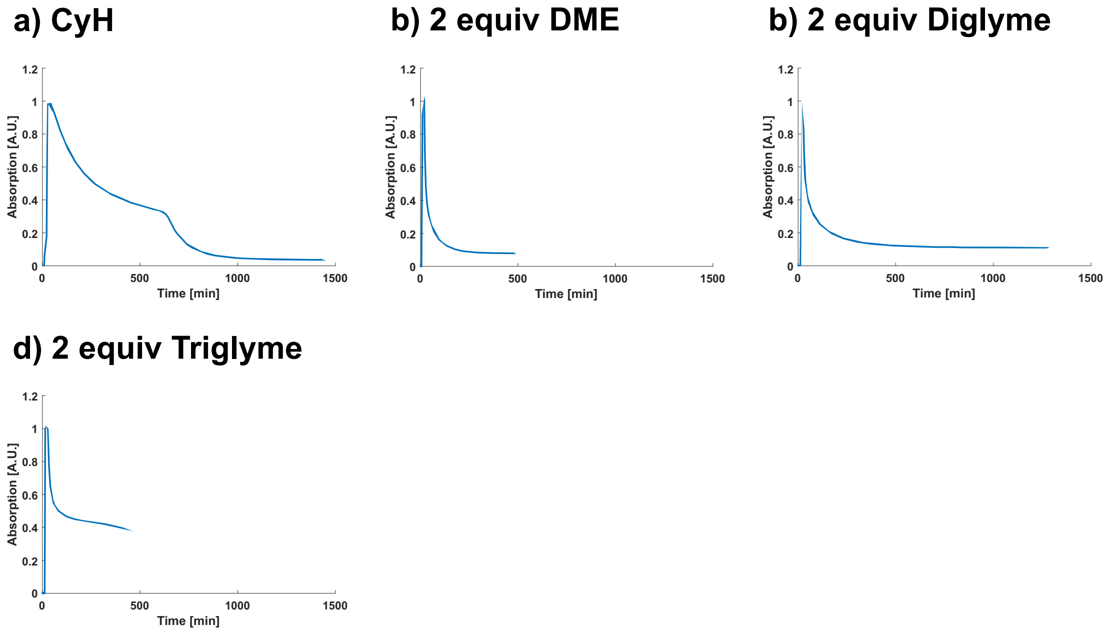

Figure S27: Tracking of the NIR absorption at $\tilde{\nu} = 6138\ \mathrm{cm}^{-1}$ during S/I copolymerizations in pure CyH (complete reaction) and in the presence of DME, diglyme and triglyme (incomplete reactions), for [Glyme]/[Li] = 2, with complete reaction characterized by absorption values approaching zero and incomplete reactions terminate at higher absorption values.

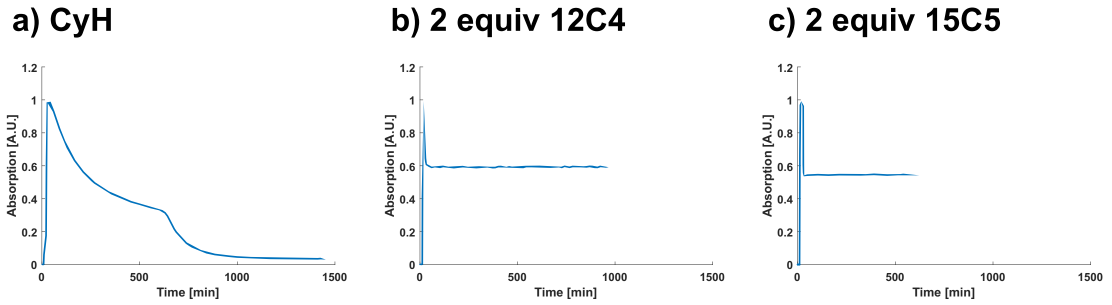

Figure S28: Tracking of the NIR absorption at $\tilde{\nu} = 6138\ \mathrm{cm}^{-1}$ during S/I copolymerizations in pure CyH (complete reaction) and in the presence of 12C4 and 15C5 (incomplete reaction) for [Crown Ether]/[Li] = 2, with complete reaction characterized by absorption values approaching zero and incomplete reactions terminate at higher absorption values.

4.3. Time-Dependent Individual Concentration Plots

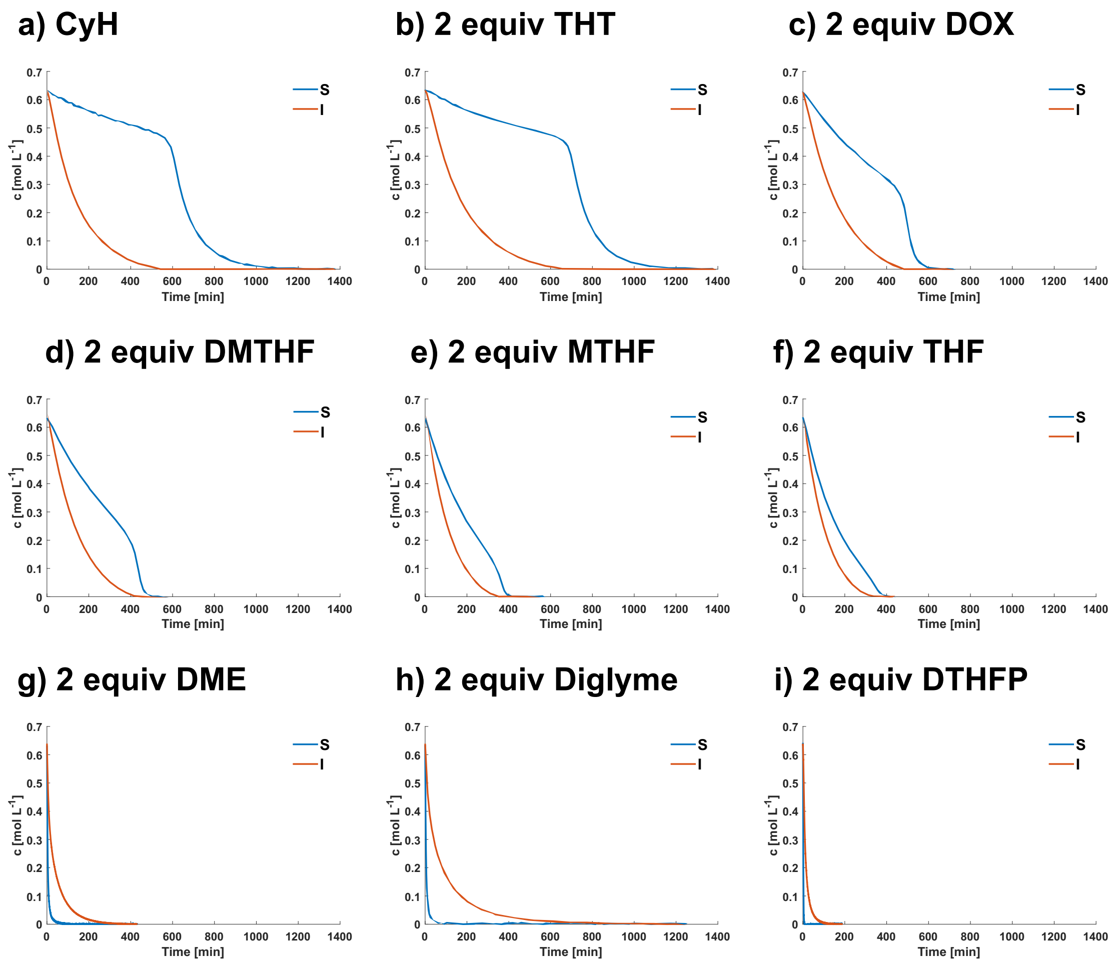

Figure S29: Individual time-conversion plots of S/I copolymerizations for [PM]/[Li] = 2.

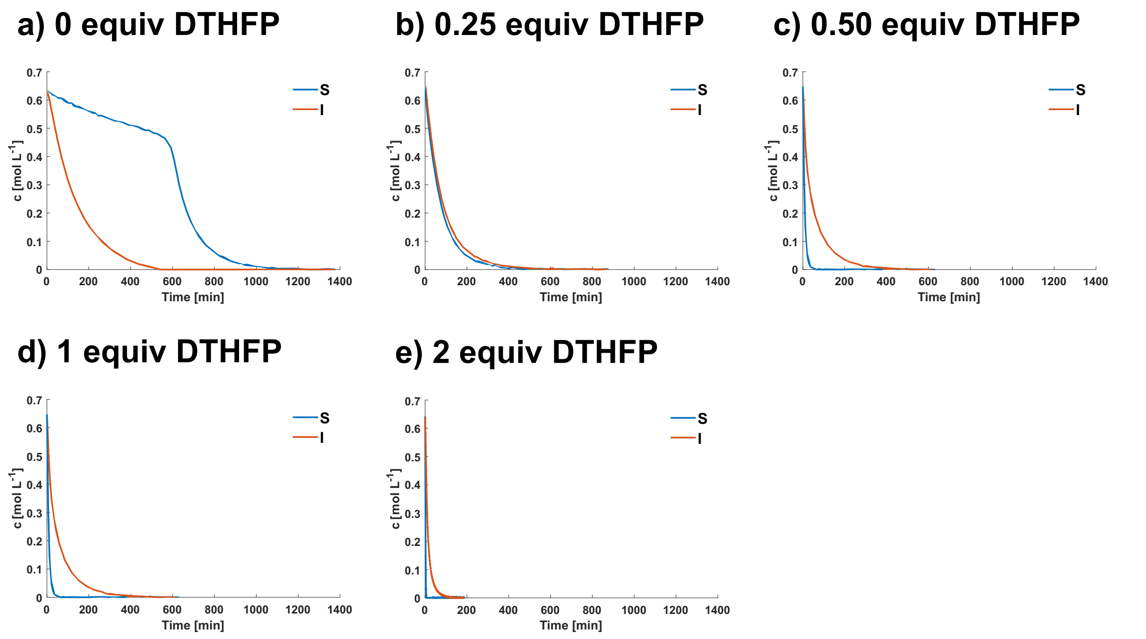

Figure S30: Individual time-conversion plots of S/I copolymerizations for various [DTHFP]/[Li] ratios.

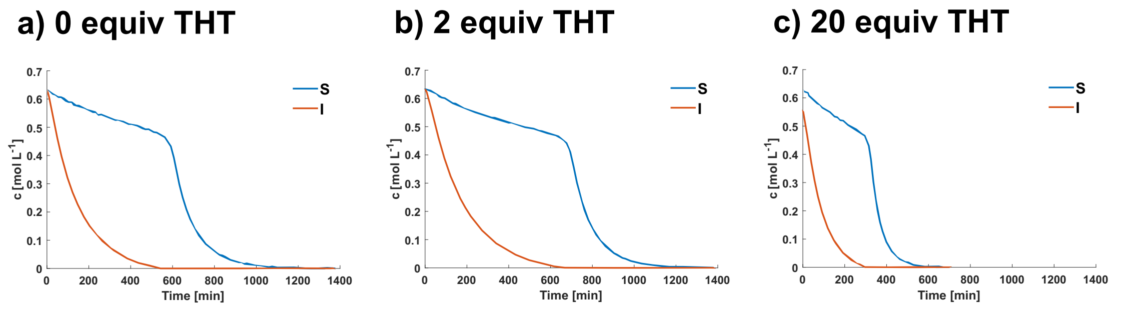

Figure S31: Individual time-conversion plots of S/I copolymerizations for various [THT]/[Li] ratios.

4.4. Individual vs Total Conversion Plots

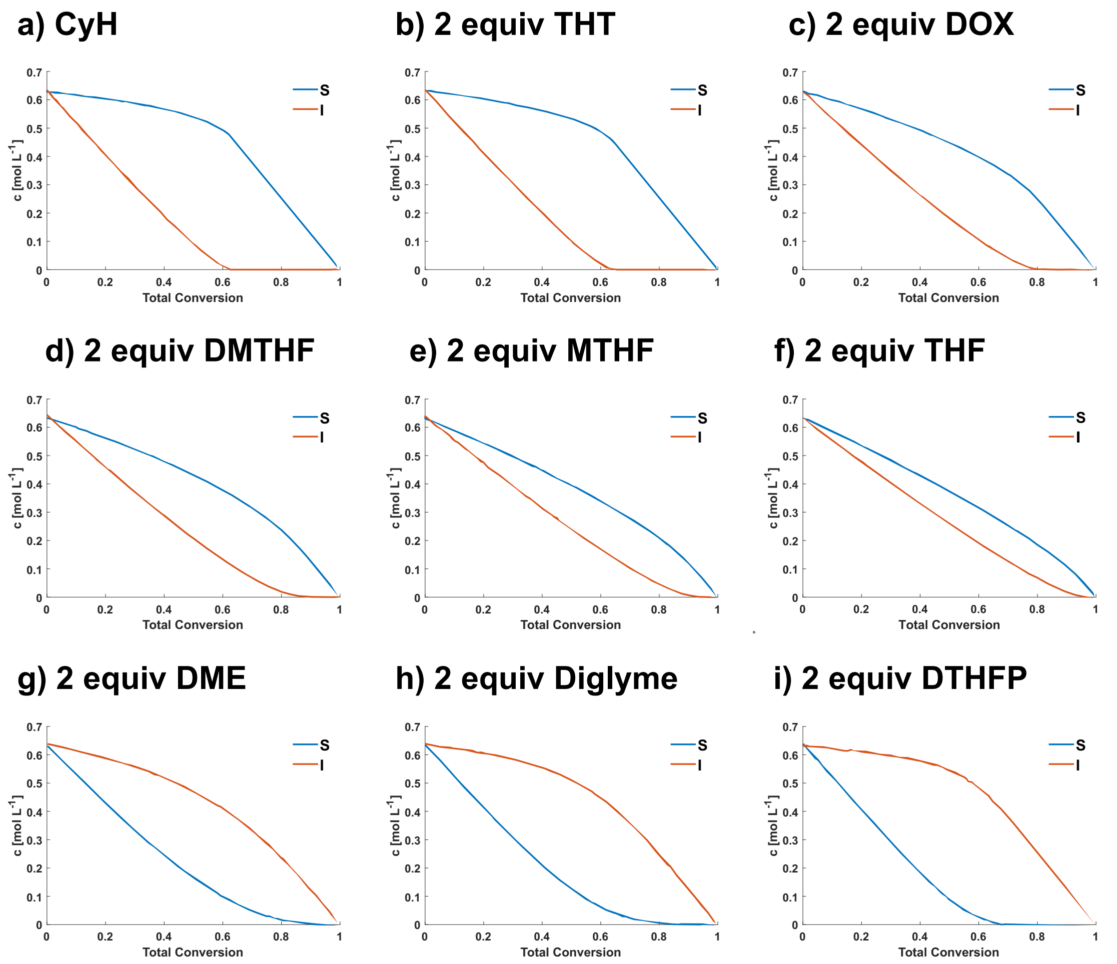

Figure S32: Individual monomer concentrations as a function of total conversion of S/I copolymerizations for [PM]/[Li] = 2.

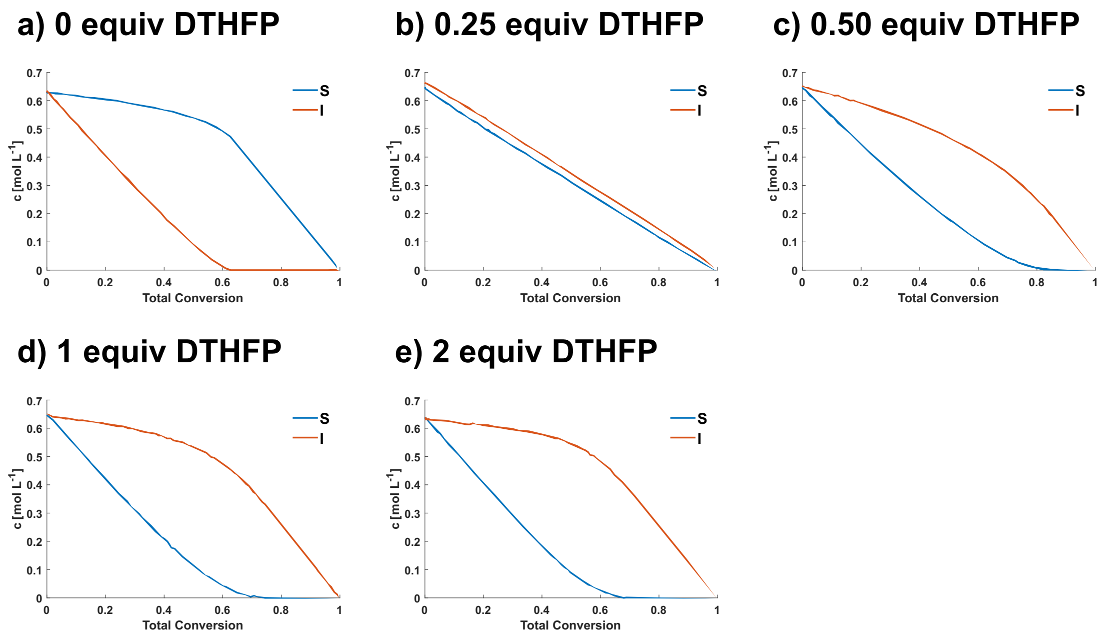

Figure S33: Individual monomer concentrations as a function of total conversion of S/I copolymerizations for various [DTHFP]/[Li] ratios.

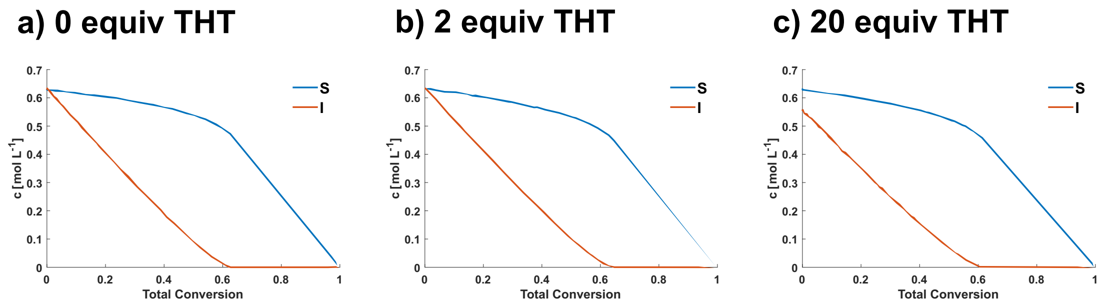

Figure S34: Individual monomer concentrations as a function of total conversion of S/I copolymerizations for various [THT]/[Li] ratios.

4.5. Fitting Method

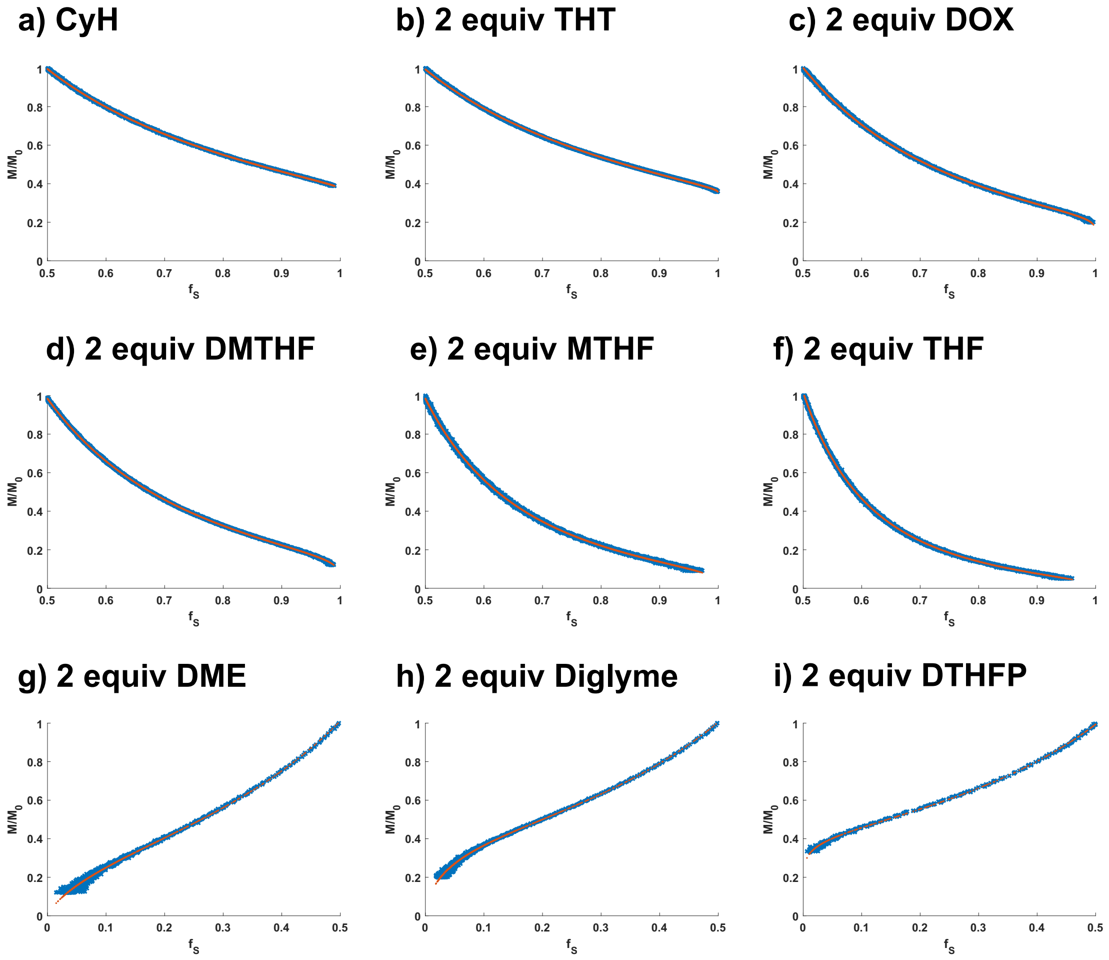

Figure S35: Meyer-Lowry fits (red lines) of S/I copolymerizations for [PM]/[Li] = 2.

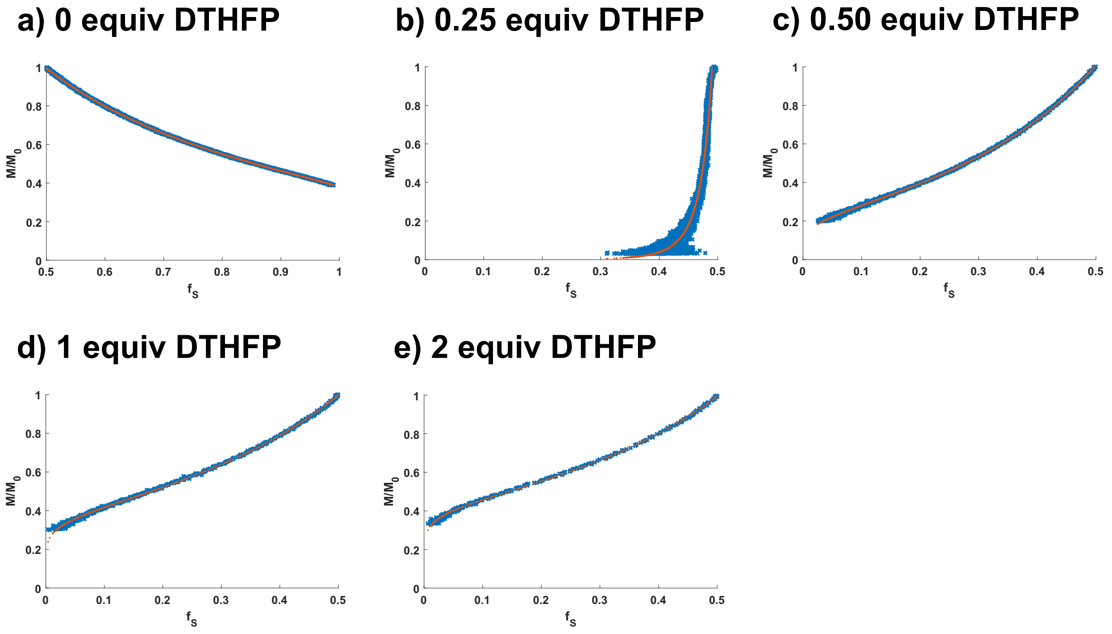

Figure S36: Meyer-Lowry fits (red lines) of S/I copolymerizations for various [DTHFP]/[Li] ratios.

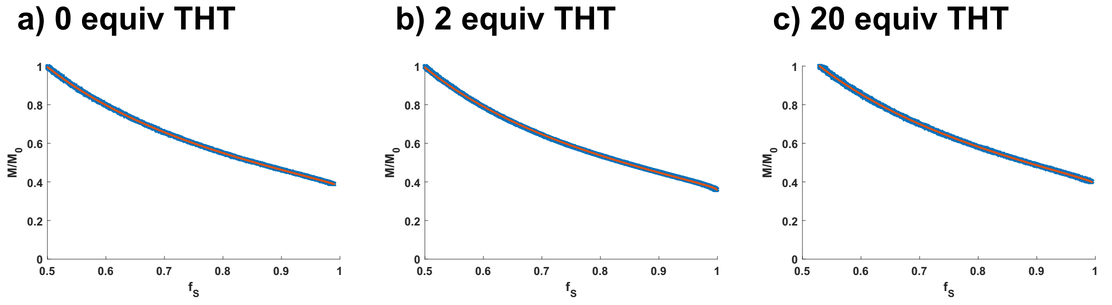

Figure S37: Meyer-Lowry fits (red lines) of S/I copolymerizations for various [THT]/[Li] ratios.

5. Investigation by NMR and DSC

5.1. Determination of Blockiness of Styrene Units *via* NMR

The blockiness of styrene units, *B*, defined as the fraction of styrene units forming contiguous sequences of two or more units within the copolymer[2], was investigated *via* ^{1}H-NMR spectroscopy (**Figure S7, S10, S13, S16, S19**) analogously to previous works[1,3,4], where this method is described in more detail.

With flattening of the gradient, the signal of the ortho-protons of PS units (h) shifts downfield and separates from the remaining aromatic signals of the PS units (i,j,h). This phenomenon can be used for a quick estimation of the blockiness.

Therefore, the integral of the total aromatic polystyrene protons (5H), I_{PS}, and the integral of the shifted ortho protons of the polystyrene block (2H), I_{SSS}, are used (**Figure S38**):
I_{PS} = 7.50–6.25 ppm (i,j,h) and I_{SSS} = 6.93–6.25 ppm (h)

The blockiness, *B*, was then calculated using **Equation (S1)**, where the respective integrals were first divided by the corresponding number of protons, followed by the determination of the ratio:

$$B = \frac{I_{SSS}/2}{I_{PS}/5} \quad \textbf{(S1)}$$

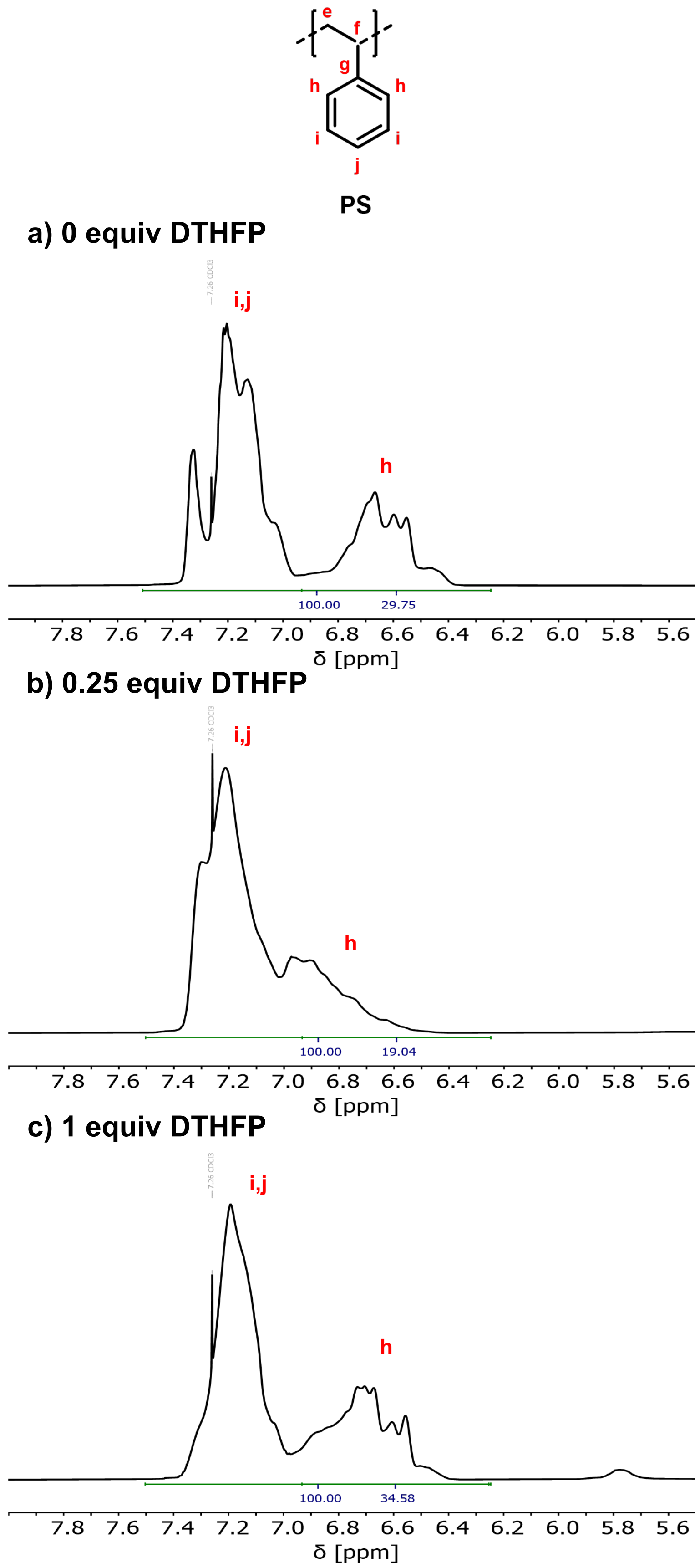

Figure S38: Integrals in ^{1}H-NMR spectra used for the calculation of the blockiness of styrene units for tapered (0 equiv DTHFP), random (0.25 equiv DTHFP), and inverse-tapered (1 equiv DTHFP) P(**I**-*co*-S) copolymers.

5.2. Determination of PI Microstructure *via* NMR

The regioisomeric composition of polyisoprene, consisting of 1,4-, 3,4- and 1,2-PI, was investigated *via* ^{1}H- (**Figures S7, S10, S13, S16, S19**) and *inverse-gated* ^{13}C-NMR spectroscopy (**Figures S8, S11, S14, S17, S20**) analogously to a previous work.[1] For each copolymer, the ^{1}H- and $^{13}C_{ig}$-NMR shifts of the olefinic signals were assigned using HSQC NMR (**Figures S9, S12, S15, S18, S21**).

The contents of 1,4-PI and 3,4-PI+1,2-PI were first quantified by *inverse-gated* ^{13}C-NMR spectroscopy. Therefore, the following integrals have been used (**Figure S39**):
$I_{1,4\text{-PI}}$ = 130.3–128.5 ppm + 125.4–122.0 ppm + 122.0–119.0 ppm (N) and
$I_{1,2\text{-PI}+3,4\text{-PI}}$ = 113.5–110.0 ppm (K', O'')

The 1,4-PI content, $C_{1,4\text{-PI}}$, was then calculated using **Equation (S2)**:

$$C_{1,4\text{-PI}} = \frac{I_{1,4\text{-PI}}}{I_{1,4\text{-PI}} + I_{1,2\text{-PI}+3,4\text{-PI}}} \qquad \textbf{(S2)}$$

The molar content of the 1,2- and 3,4-PI units, $C_{1,2\text{-PI}+3,4\text{-PI}}$, is given by **Equation (S3)**:

$$C_{1,2\text{-PI}+3,4\text{-PI}} = 1 - C_{1,4\text{-PI}} = \frac{I_{1,2\text{-PI}+3,4\text{-PI}}}{I_{1,4\text{-PI}} + I_{1,2\text{-PI}+3,4\text{-PI}}} \qquad \textbf{(S3)}$$

Subsequently, the molar content of 1,2-PI, $C_{1,2\text{-PI}}$, and 3,4-PI, $C_{3,4\text{-PI}}$, was determined by ^{1}H-NMR spectroscopy using **Equation (S4)** and the following integrals (**Figure S40**):
$I_{1,2\text{-PI}}$ = 6.00–5.30 ppm (n'') and $I_{\text{total}} = I_{1,4\text{-PI}+3,4\text{-PI}+1,2\text{-PI}}$ = 5.25–4.10 ppm (n, k', o'')

$$C_{1,2\text{-PI}} = \frac{I_{1,2\text{-PI}}}{I_{\text{PI,total}}/H_{\text{olefinic,average}}} = \frac{I_{1,2\text{-PI}} \cdot H_{\text{olefinic,average}}}{I_{\text{PI,total}}} = \frac{DP_{1,2\text{-PI}}}{DP_{\text{PI,total}}} \qquad \textbf{(S4)}$$

The average number of olefinic protons, $H_{\text{olefinic,average}}$, was calculated as followed (**Equation (S5)**):

$$H_{\text{olefinic,average}} = C_{1,4\text{-PI}} \cdot H_{1,4\text{-PI}} + C_{1,2\text{-PI}+3,4\text{-PI}} \cdot H_{1,2\text{-PI}+3,4\text{-PI}} \qquad \textbf{(S5)}$$

Here, the number of protons of the integrated signals, which were used, are $H_{1,4\text{-PI}} = 1$ (n) and $H_{1,2\text{-PI}+3,4\text{-PI}} = 2$ (k', o'').
Lastly, with the previously calculated molar content of the 1,4-PI and 1,2-PI units, the molar content of the 3,4-PI units can be determined (**Equation (S6)**):

$$C_{3,4\text{-PI}} = 1 - C_{1,4\text{-PI}} - C_{1,2\text{-PI}} \qquad \textbf{(S6)}$$

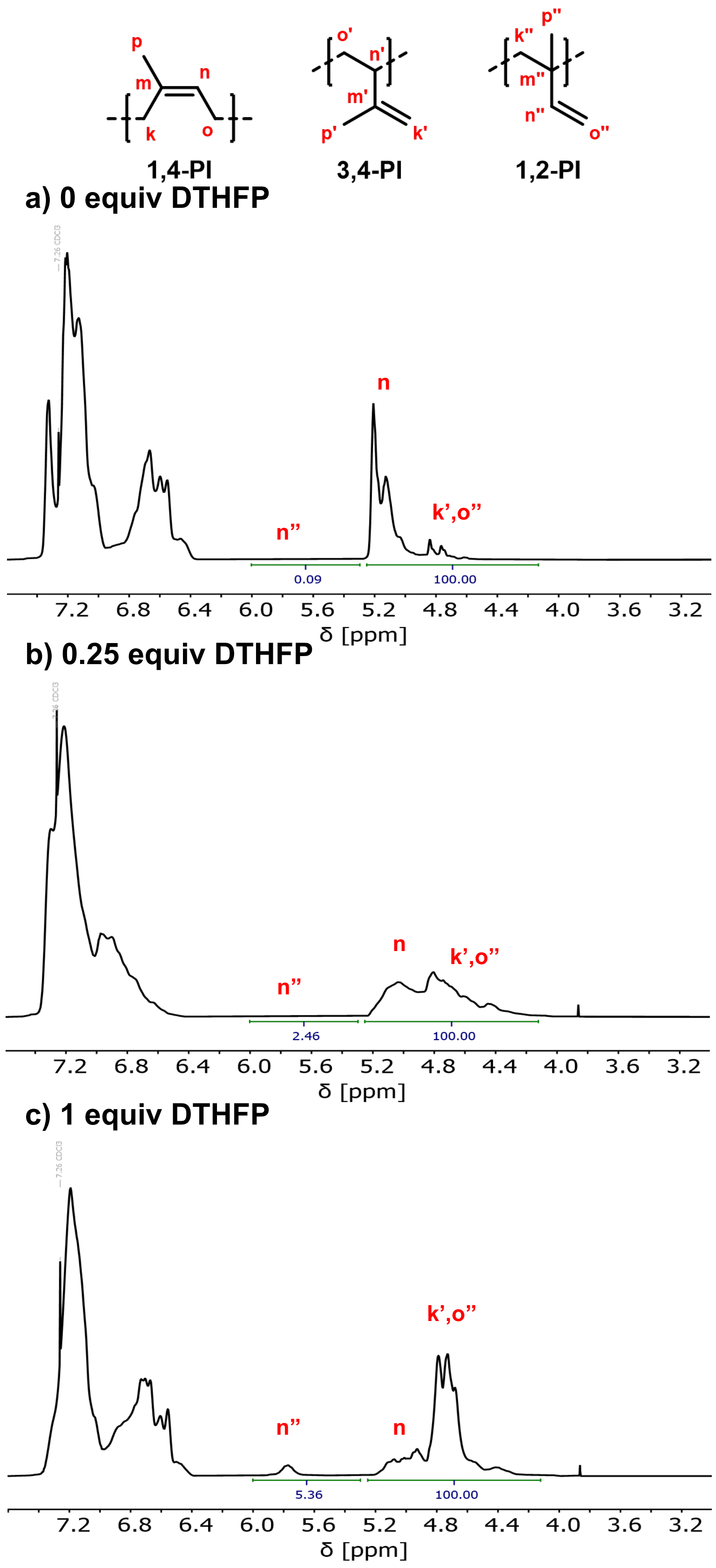

Figure S39: Integrals in ^{1}H-NMR spectra used for the calculation of the PI microstructure composition for tapered (0 equiv DTHFP), random (0.25 equiv DTHFP), and inverse-tapered (1 equiv DTHFP) P(I-*co*-S) copolymers.

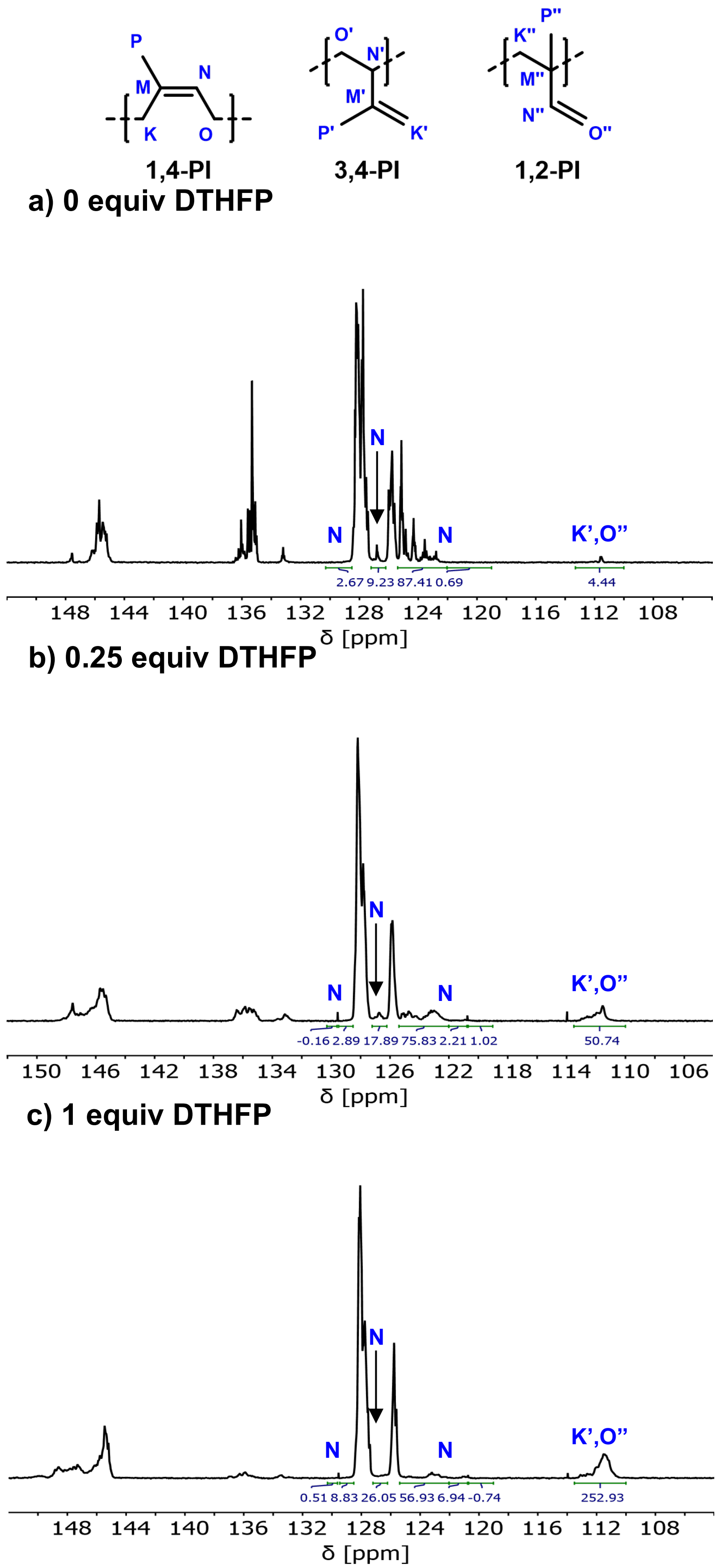

Figure S40: Integrals in *inverse-gated* ^{13}C-NMR spectra used for the calculation of the PI microstructure composition for tapered (0 equiv DTHFP), random (0.25 equiv DTHFP), and inverse-tapered (1 equiv DTHFP) P(I-*co*-S) copolymers.

5.3. Determination of Thermal Properties *via* DSC

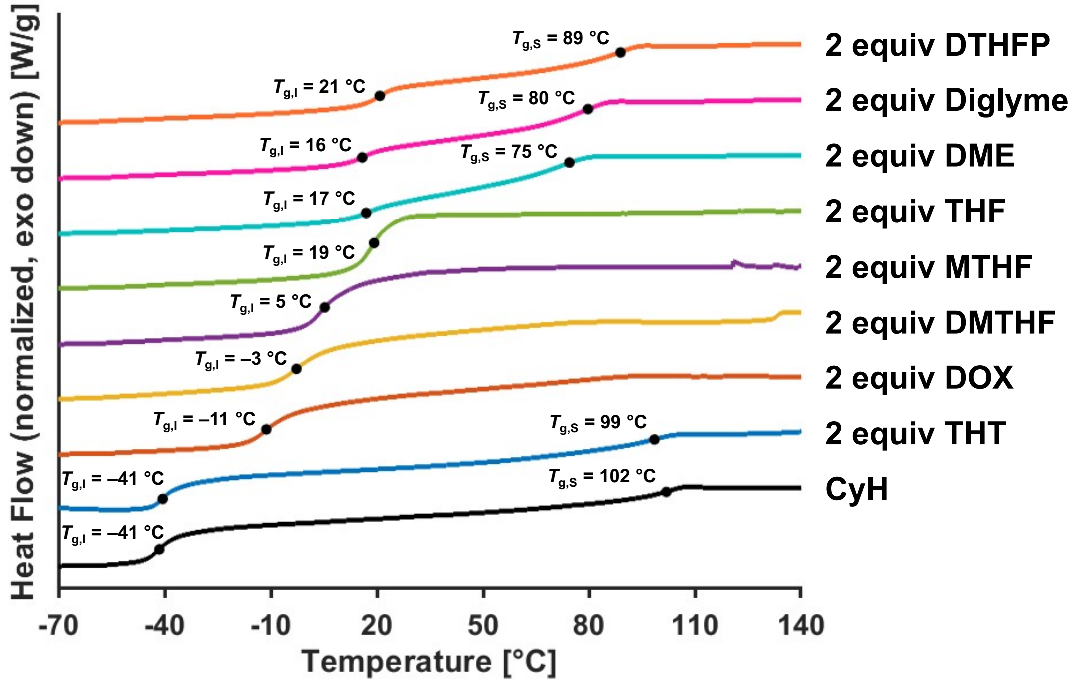

Figure S41: Second heating curve of P(I-*co*-S) copolymers for [PM]/[Li] = 2.

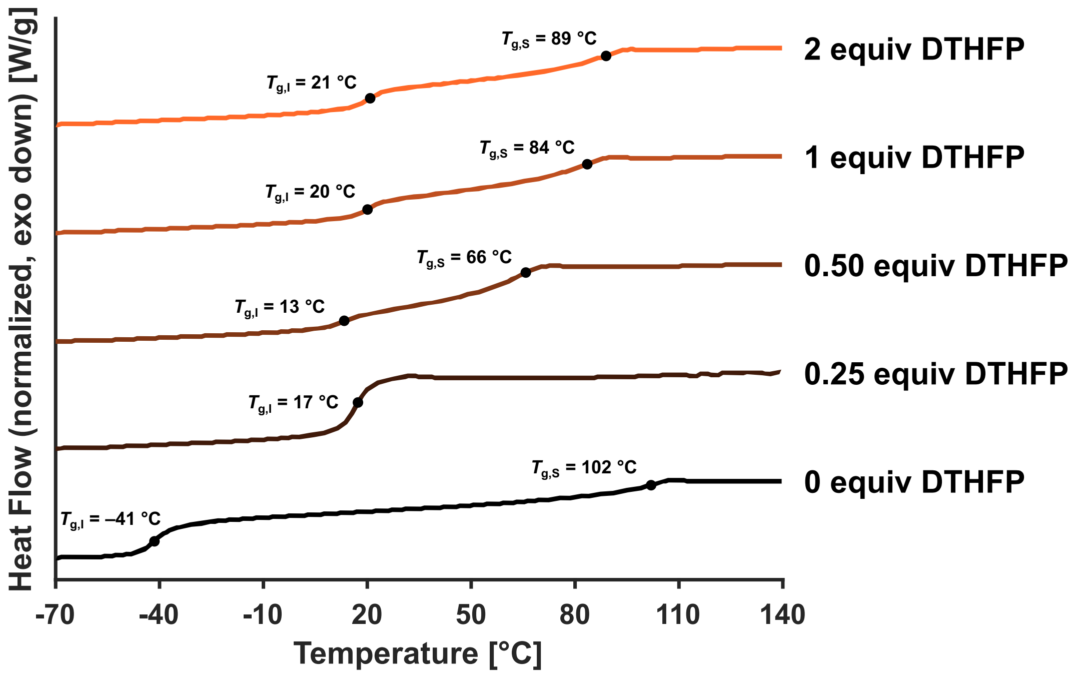

Figure S42: Second heating curve of P(I-*co*-S) copolymers for various [DTHFP]/[Li] ratios.

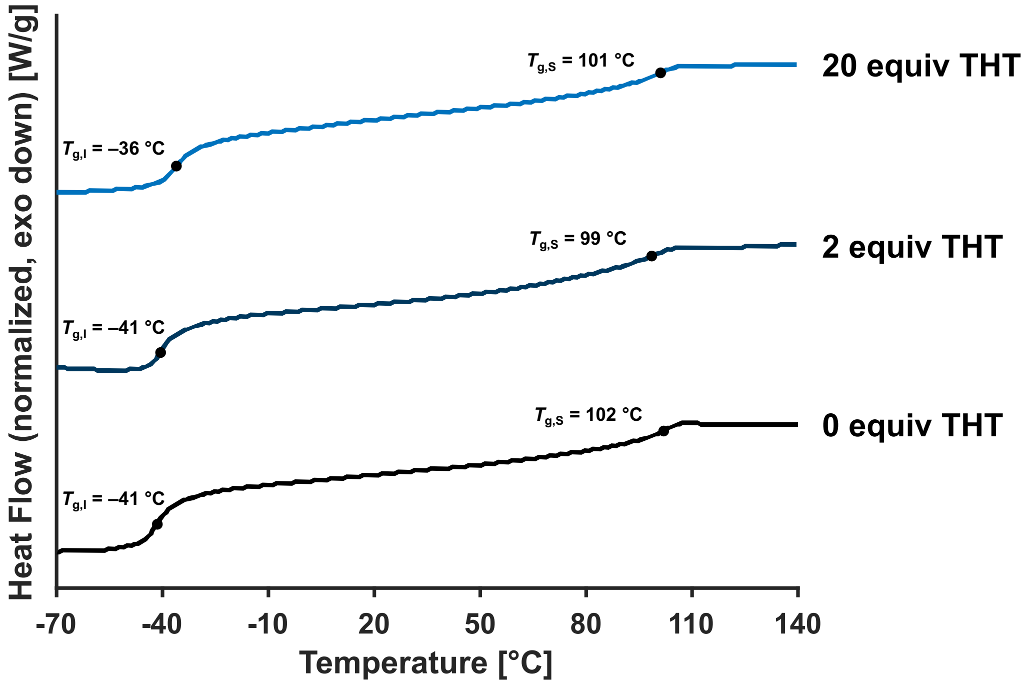

Figure S43: Second heating curve of P(I-*co*-S) copolymers for various [THT]/[Li] ratios.

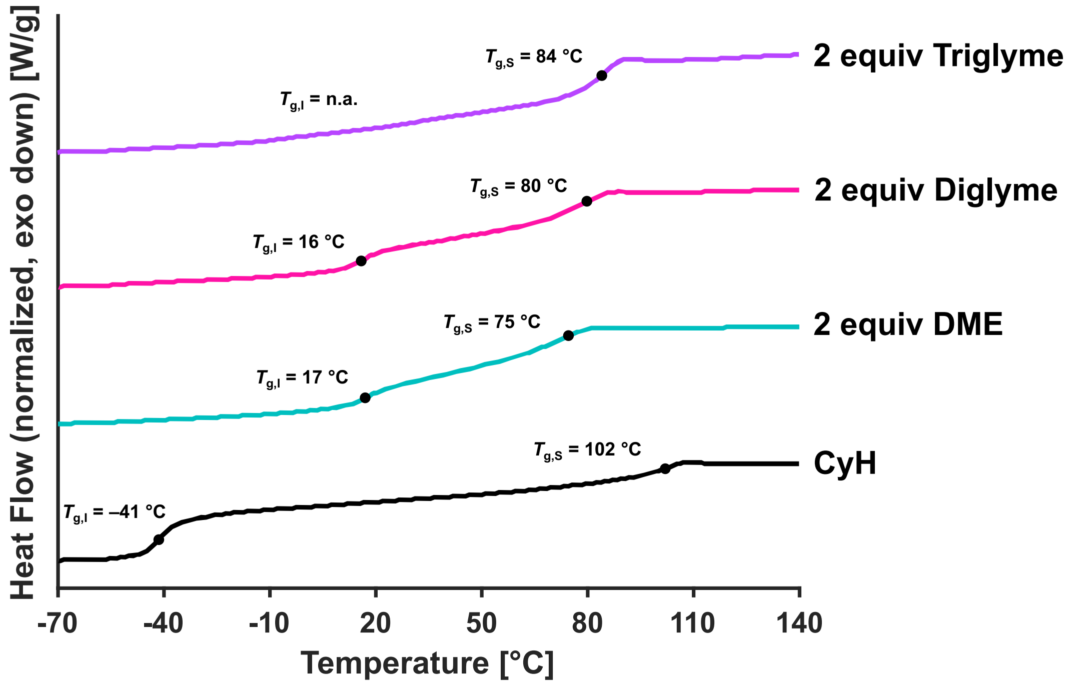

Figure S44: Second heating curve of P(I-*co*-S) copolymers for [Glyme]/[Li] = 2.

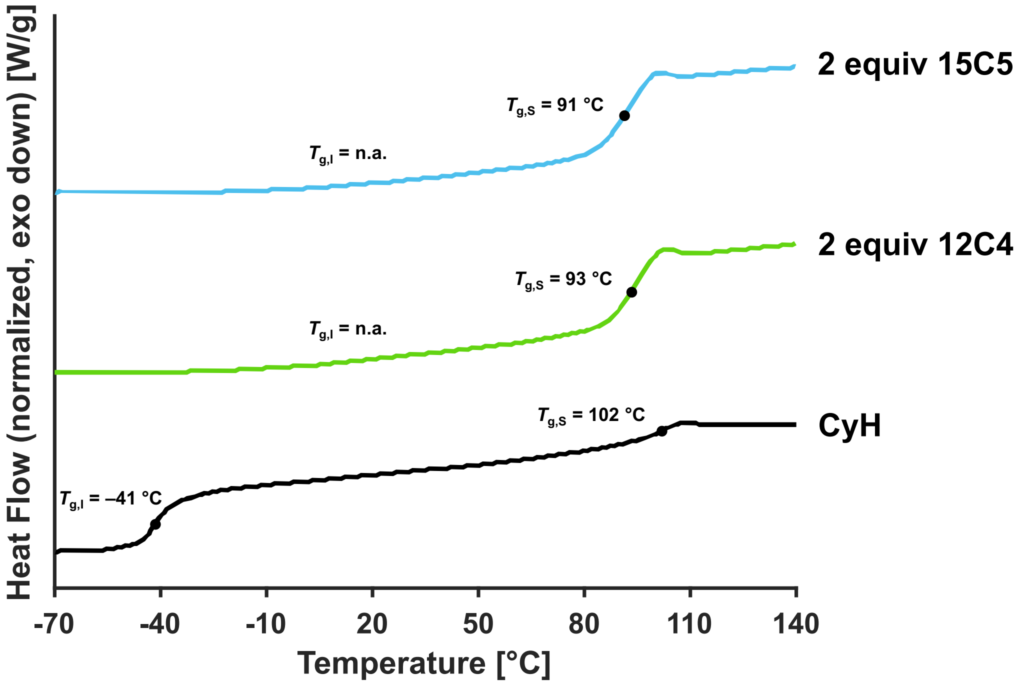

Figure S45: Second heating curve of P(I-*co*-S) copolymers for [Crown Ether]/[Li] = 2.

6. Visualization of Experimental Results

6.1. Copolymer Composition Diagrams

The molar composition diagrams were calculated using the determined reactivity ratios:

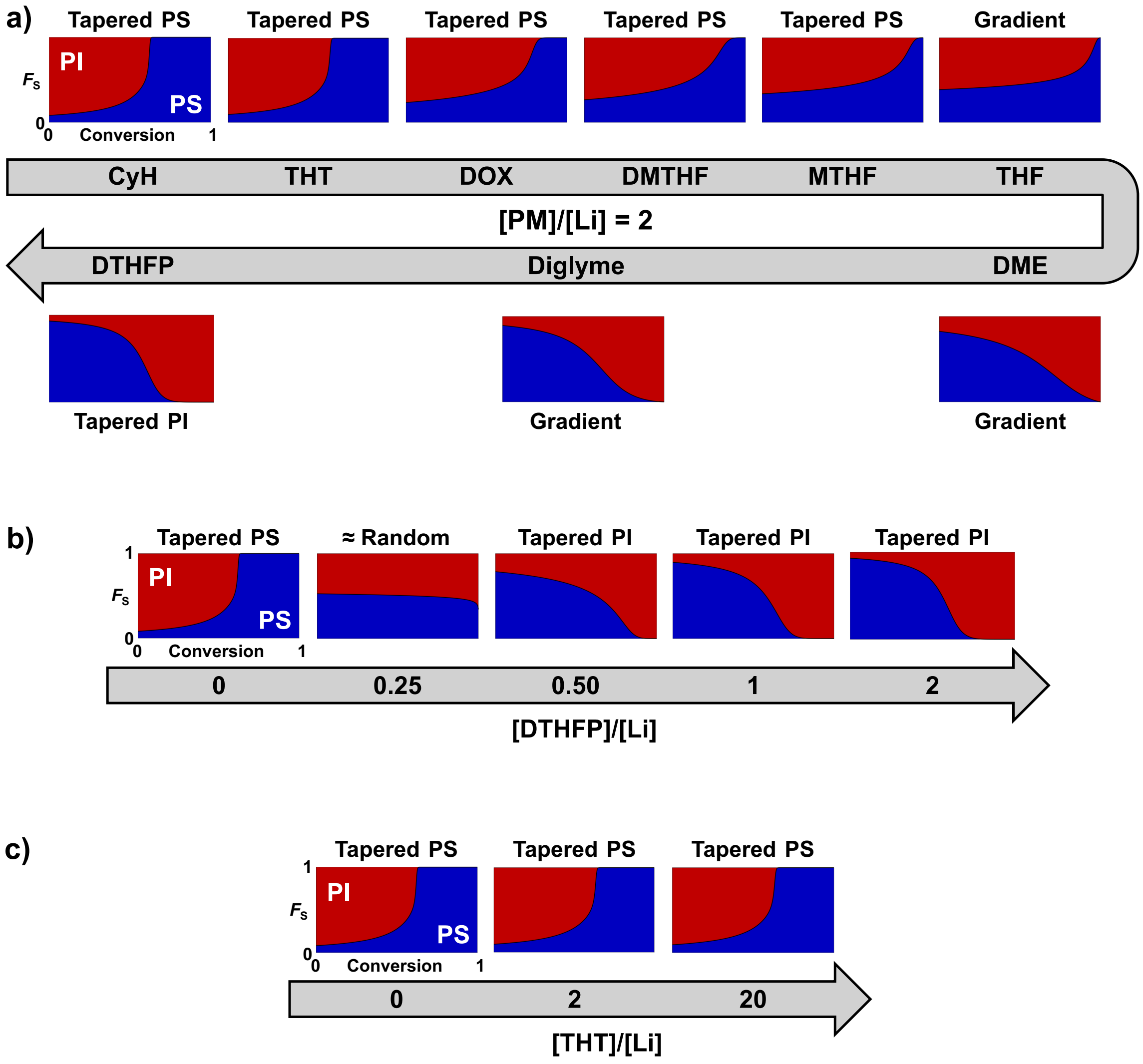

Figure S46: Molar composition profiles of P(I-*co*-S) copolymers for (a) [PM]/[Li] = 2, (b) various [DTHFP]/[Li] ratios and (c) various [THT]/[Li] ratios.

The volume composition diagrams were calculated using the molar mass of the monomers and the densities of the homopolymers ($\rho_{PI} = 0.83\ \mathrm{g/cm^3}$ and $\rho_{PS} = 0.97\ \mathrm{g/cm^3}$)[5]:

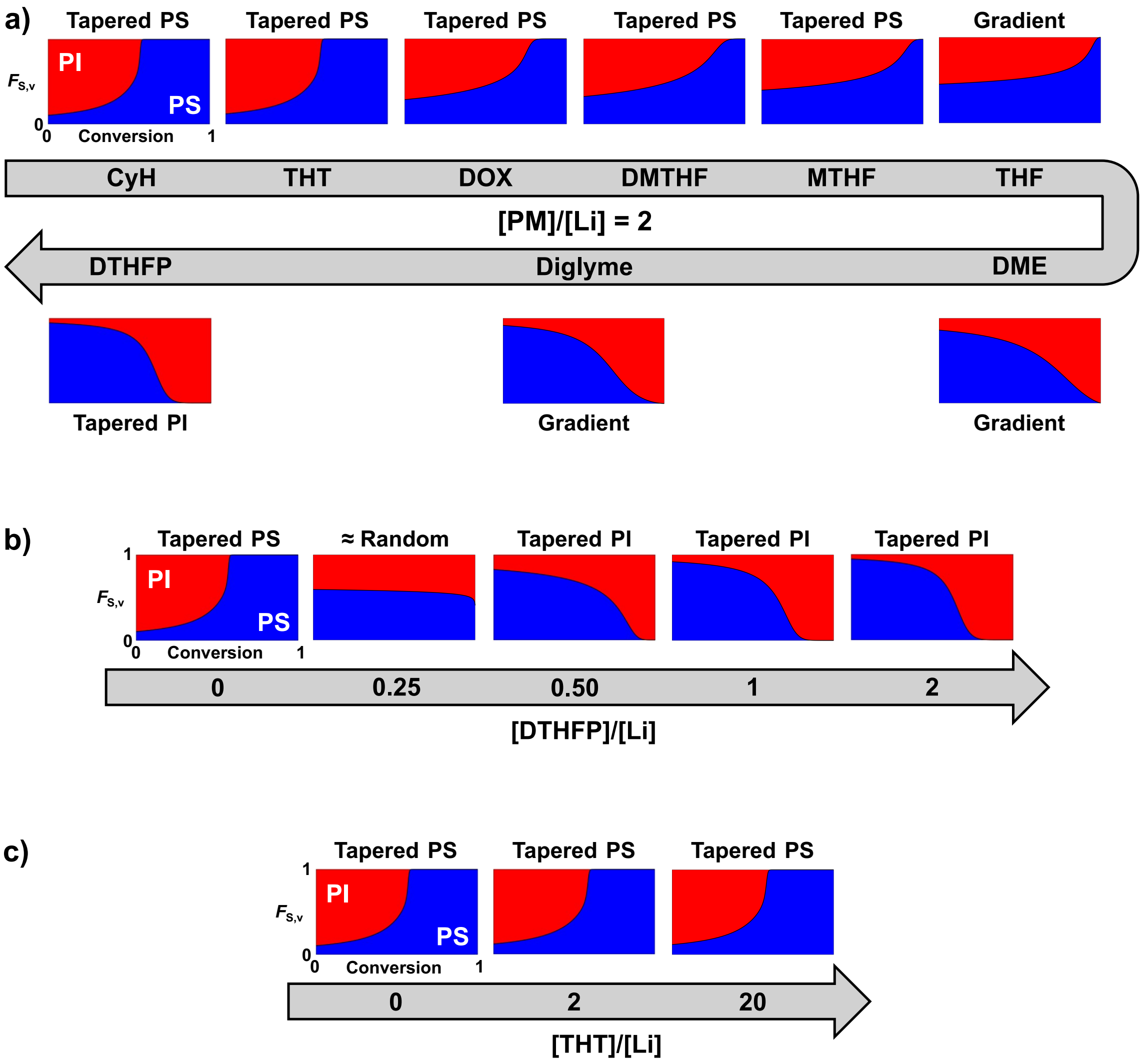

Figure S47: Volume composition profiles of P(I-*co*-S) copolymers for (a) [PM]/[Li] = 2, (b) various [DTHFP]/[Li] ratios and c) various [THT]/[Li] ratios.

6.2. Plots of Individual Time-Conversion, Individual *vs* Total Conversion, and Reactivity Ratios for THT Series as well as Plots of (Nomalized) Half-lives

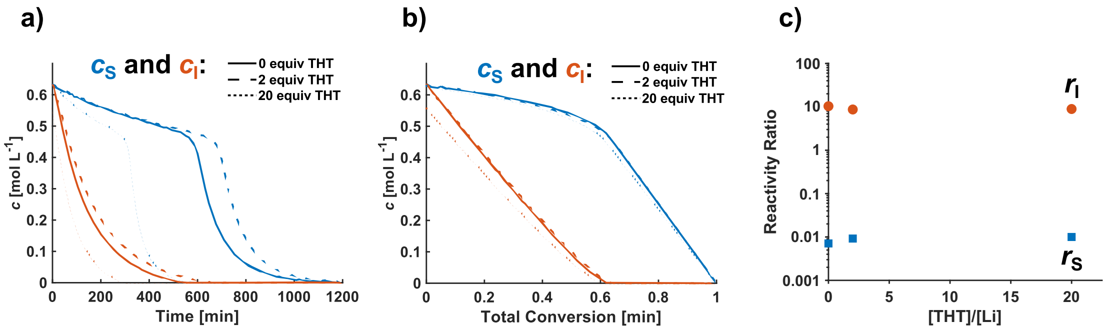

Figure S48: (a) Individual time-conversion plots, (b) selective individual *vs* total conversion plots and (c) reactivity ratios of S/I copolymerizations for for various [THT]/[Li] ratios.

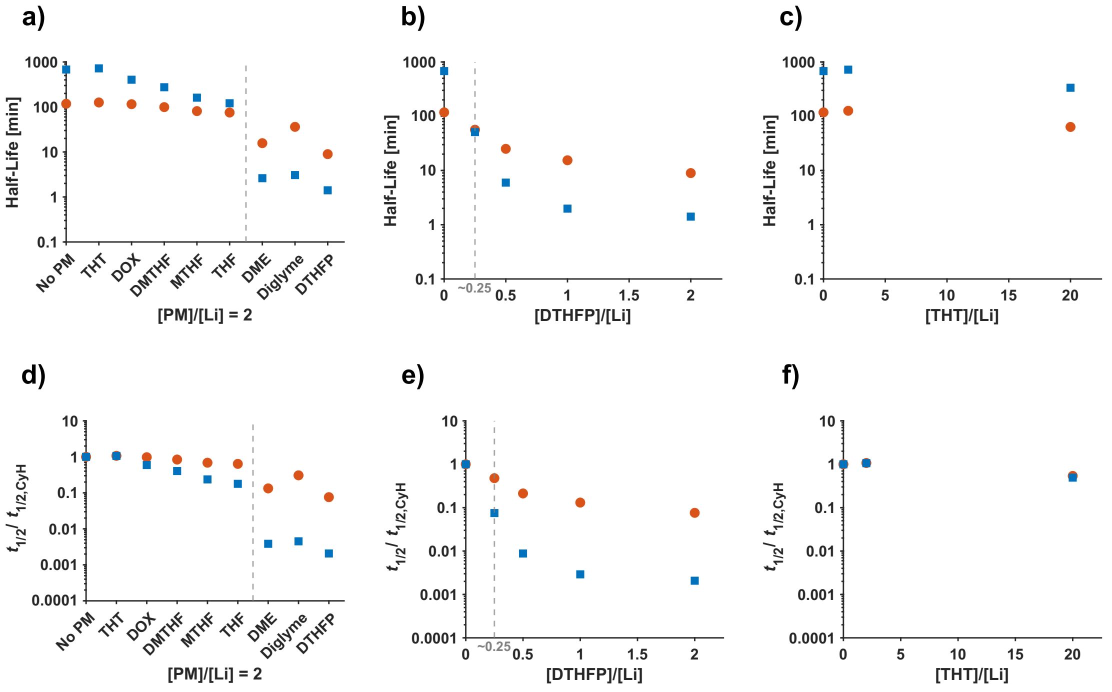

Figure S49: Half-lives (top row) and half-lives normalized by the half-lives in pure cyclohexane (bottom row) of isoprene and styrene for (a, d) [PM]/[Li] = 2, (b, e) various [DTHFP]/[Li] ratios and (c, f) various [THT]/[Li] ratios; with dashed lines separating monodentate PM from bi- and tridentate PMs (left column) or indicating a random architecture for 0.25 equiv DTHFP (middle column).

6.3. Relation of Reactivity Ratios and Donor Number for Monodentate Polar Modifiers

The Gutmann donor number (*DN*) is an empirical measure of the Lewis basicity (donor-strength) of a solvent. *DN* is defined as the positive value of the negative enthalpy ($-\Delta H$) of adduct formation between the solvent and a standard Lewis acid (acceptor-halide), typically antimony(V) pentachloride ($SbCl_5$), in a non-coordinating (inert) solvent as 1,2-dichloroethane. It is expressed in kcal mol^{-1} and reflects the electron-pair donating ability of the solvent.[6]

Determined reactivity ratios and corresponding donor numbers of the monodentate polar modifiers (DOX, MTHF, and THF) were used for the following fit:

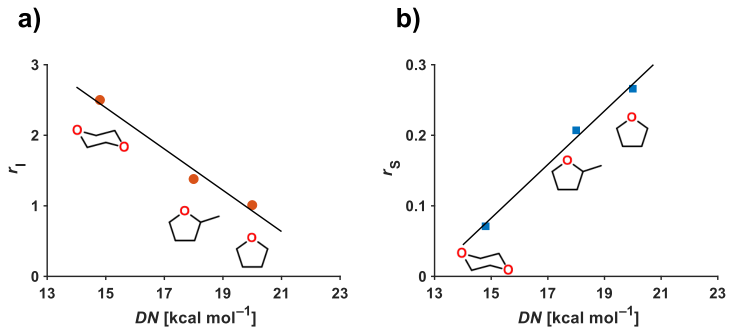

Figure S50: Reactivity ratios *vs* donor number plots of (a) isoprene and (b) styrene for the monodentate polar modifiers DOX, MTHF and THF.

No Gutmann donor number has been reported for DMTHF. Based on structural similarity, it is reasonable to assume that its *DN* lies between that of DOX (*DN* = 14.8 kcal mol^{-1})[7] and MTHF (*DN* = 18 kcal mol^{-1})[8]. This assumption is consistent with the general trend of decreasing donor number upon methyl substitution of THF (*DN* = 20 kcal mol^{-1})[7].

6.4. Plots of Glass Transition Temperature, Blockiness, and PI Microstructure for Additional Polar Modifier Series

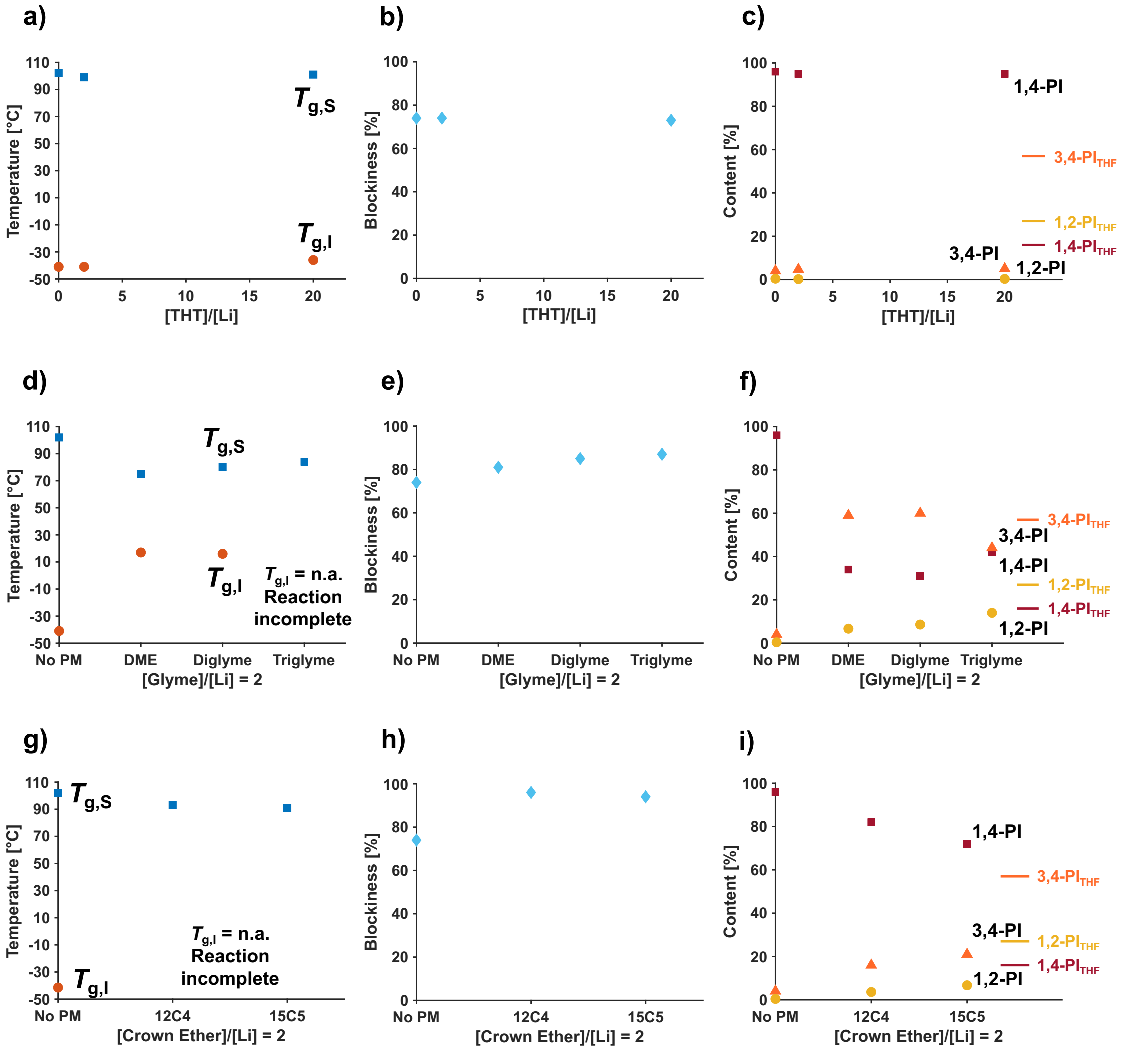

Figure S51: (a,d,g) glass transition temperature, (b,e,h) blockiness and (c,f,i) PI microstructure of P(I-*co*-S) copolymers for various [THT]/[Li] ratios (top row), [Glyme]/[Li] = 2 (middle row) and [Crown Ether]/[Li] = 2 (bottom row).

7. References

(1) Steube, M.; Johann, T.; Hübner, H.; Koch, M.; Dinh, T.; Gallei, M.; Floudas, G.; Frey, H.; Müller, A. H. E. Tetrahydrofuran: More than a "Randomizer" in the Living Anionic Copolymerization of Styrene and Isoprene: Kinetics, Microstructures, Morphologies, and Mechanical Properties. *Macromolecules* **2020**, *53* (13), 5512–5527. DOI: 10.1021/acs.macromol.0c01022.

(2) Mochel, V. D. Nuclear Magnetic Resonance-Analog Computer Method for "Block Styrene". *Macromolecules* **1969**, *2* (5), 537–540. DOI: 10.1021/ma60011a017.

(3) Fuchs, D. A. H.; Hübner, H.; Kraus, T.; Niebuur, B.-J.; Gallei, M.; Frey, H.; Müller, A. H. E. The Effect of THF and the Chelating Modifier DTHFP on the Copolymerisation of β-Myrcene and Styrene: Kinetics, Microstructures, Morphologies, and Mechanical Properties. *Polym. Chem.* **2021**, *12* (32), 4632–4642. DOI: 10.1039/D1PY00791B.

(4) Fuchs, D. A. H.; Frey, H.; Müller, A. H. E. Dramatic Effect of Alkali Metal Alkoxides on the Anionic Copolymerization of Styrene and Isoprene. *Macromolecules* **2025**, *58* (13), 6854–6864. DOI: 10.1021/acs.macromol.5c00975.

(5) Fetters, L. J.; Lohse, D. J.; Richter, D.; Witten, T. A.; Zirkel, A. Connection between Polymer Molecular Weight, Density, Chain Dimensions, and Melt Viscoelastic Properties. *Macromolecules* **1994**, *27* (17), 4639–4647. DOI: 10.1021/ma00095a001.

(6) Gutmann, V.; Wychera, E. Coordination Reactions in Non Aqueous Solutions - The Role of the Donor Strength. *Inorg. Nucl. Chem. Letters* **1966**, *2* (9), 257–260. DOI: 10.1016/0020-1650(66)80056-9.

(7) Gutmann, V. Empirical Parameters for Donor and Acceptor Properties of Solvents. *Electrochim. Acta* **1976**, *21* (9), 661–670. DOI: 10.1016/0013-4686(76)85034-7.

(8) Gutmann, V. Solvent Effects on the Reactivities of Organometallic Compounds. *Coord. Chem. Rev.* **1976**, *18* (2), 225–255. DOI: 10.1016/S0010-8545(00)82045-7.
